# Impact of autocatalytic chemical reaction in an Ostwald-de-Waele nanofluid flow past a rotating disk with heterogeneous catalysis

**DOI:** 10.1038/s41598-021-94918-7

**Published:** 2021-07-30

**Authors:** Bai Yu, Muhammad Ramzan, Saima Riasat, Seifedine Kadry, Yu-Ming Chu, M. Y. Malik

**Affiliations:** 1 School of Science, Beijing University of Civil Engineering and Architecture, Beijing, 100044 People’s Republic of China; 2 Beijing Key Laboratory of Functional Materials for Building Structure and Environment Remediation, Beijing University of Civil Engineering and Architecture, Beijing, 100044 People’s Republic of China; 3 Department of Computer Science, Bahria University, Islamabad, 44000 Pakistan; 4 Faculty of Applied Computing and Technology, Noroff University College, Kristiansand, Norway; 5 Department of Mathematics, Huzhou University, Huzhou, 313000 People’s Republic of China; 6 Hunan Provincial Key Laboratory of Mathematical Modeling and Analysis in Engineering, Changsha University of Science and Technology, Changsha, 410114 People’s Republic of China; 7 Department of Mathematics, College of Sciences, King Khalid University, Abha, 61413 Kingdom of Saudi Arabia

**Keywords:** Software, Mechanical engineering

## Abstract

The nanofluids owing to their alluring attributes like enhanced thermal conductivity and better heat transfer characteristics have a vast variety of applications ranging from space technology to nuclear reactors etc. The present study highlights the Ostwald-de-Waele nanofluid flow past a rotating disk of variable thickness in a porous medium with a melting heat transfer phenomenon. The surface catalyzed reaction is added to the homogeneous-heterogeneous reaction that triggers the rate of the chemical reaction. The added feature of the variable thermal conductivity and the viscosity instead of their constant values also boosts the novelty of the undertaken problem. The modeled problem is erected in the form of a system of partial differential equations. Engaging similarity transformation, the set of ordinary differential equations are obtained. The coupled equations are numerically solved by using the bvp4c built-in MATLAB function. The drag coefficient and Nusselt number are plotted for arising parameters. The results revealed that increasing surface catalyzed parameter causes a decline in thermal profile more efficiently. Further, the power-law index is more influential than the variable thickness disk index. The numerical results show that variations in dimensionless thickness coefficient do not make any effect. However, increasing power-law index causing an upsurge in radial, axial, tangential, velocities, and thermal profile.

## Introduction

The customary fluids like oil and water are poor heat transfer liquids as they hold low thermal conductivities. As the role of thermal conductivity is vital in heat transfer processes, therefore, a variety of methods are devised to enhance the thermal conductivity. The efficient technique for the enhancement of thermal conductivity is to insert the nanoparticles into the base liquids. The nanoparticles may be from metals, oxides, nitrides, or carbides. The nanofluids with unique characteristics have wide-ranging applications including fuel cells, hybrid-powered engines, and pharmaceuticals, etc. The novel notion of nanofluids is introduced by Choi^1^ in 1995. Later, many investigations have been reported^2–6^. Lately, Ram and Kumar^7^ analyzed the fluid flow past a rotating disk with viscosity as a function of temperature. Rashidi et al.^8^ investigated the fluid flow past a rotating spongy disk by using numerical method. They focused on the temperature-dependent viscosity, density, and thermal conductivity. Sheikholeslami et al.^9^ examined the three-dimensional condensation nanofluid thin-film flow by employing the effects of thermophoresis and Brownian motion with normalized thickness over an inclined rotating disk. Bachok et al.^10^ considered the Maxwell–Garnett model and the Patel model to study the impact of effective thermal conductivity of nanofluid flow past a rotating porous disk. Kendoush^11^ obtained the similarity solution to visualize the heat transfer rate for rotational and flow Reynolds number. Turkyilmazoglu^12^ performed the boundary layer flow analysis by considering copper and Aluminum oxide. The main conclusion of their finding was that copper has much heat transfer rate than Aluminum oxide. The study of heat augmentation by employing nanofluid has been the main concern of researchers^13–17^. Hayat et al.^18^ analyzed the increasing effect of heat transfer rate for elevating nanoparticle volume fraction for MHD nanofluid flow due to rotating disk. Various researchers have put much effort into identifying the beneficial properties of nanofluid^19–24^.

Presently the researchers are more interested in studying non-Newtonian fluids for their abundant industrial and engineering applications in comparison to the Newtonian fluid. Therefore, the class of Non-Newtonian fluid has gained considerable attention from many researchers. Mitschka et al.^25,26^ analyzed the power-law Non-Newtonian fluids to derive the relation for the frictional resistance of the disk. Andreson et al.^27^ investigated that boundary layer thickness decreases for diminishing values of power-law index. They eliminated the ambiguity in the previous results obtained for shear-thinning fluids by obtaining the results for shear thickening fluids. Attia^28^ studied the heat transfer process of unsteady Reiner-Rivlin flow past a rotating disk. Sahoo^29^ studied the Von Karman flow of non-Newtonian fluid. Ahmadpour and Sadeghy^30^ discussed the swirling flow of Bingham fluid. They studied the impact of yield stress on boundary layer thickness and volumetric flow rate. Griffiths^31^ studied the power-law and Bingham fluid due to rotating disks. Griffiths et al.^32^ studied the neutral curve for power-law fluids on a rotating disk. Lin et al.^33^ considered the convective heat transfer phenomenon in power-law fluids along with an inclined plate. They concluded that the heat transfer process is highly dependent on the power-law exponent. Following their models, more thermal conductivity models were addressed^34,35^. Ming et al.^36^ evaluated the steady heat transfer phenomenon of power-law fluid past a rotating disk. They studied the significant impacts of the power-law index on thermal and radial velocity profiles.

Due to abundant civil, aeronautical, mechanical applications, the deformable or elements of variable thickness have been taken into consideration. Shufrin^37^ analyzed the stability of deformable plates. To improve the utilization ability and to reduce the weight of structural elements, the plate elements have been considered. Fang et al.^38^ explored the sheet of variable thickness with power-law surface velocity. Stretching sheets of variable thickness have been reexamined by obtaining the dual solution in a thermal diffusive flow^39^. Hayat et al.^40^ investigated the same problem by considering the Cattaneo-Christov heat flux model. Wahed et al.^41^ deliberated the research on nanofluid flow with non-linear velocity over a moving surface of varying thickness. Li et al.^42^ studied the nanofluid flow past a rotating disk of variable thickness for power-law fluid. Xun et al.^43^ studied the Ostwald-de Waele fluid past a rotating disk of variable thickness. Nanofluid flow past a rotating disk with variable thickness influenced by melting heat transfer is examined by Hayat et al.^44^.

The homogeneous-heterogeneous reaction is executed in the presence of a catalyst. The rate of the chemical reaction is enhanced in the attendance of a catalyst. However, in the presence of surface catalyzed chemical reaction the rate of reaction is more accelerated. Chaudhary and Merkin^45^ were the pioneers who developed the model for homogeneous-heterogeneous reactions. Recently homogeneous heterogeneous in the disk problem has been studied copiously^46,47^. Liu et al.^48^ studied the impact of the surface catalyzed parameter by considering porous media. Hayat et al.^49^ studied the Darcy-Forchheimer nanofluid flow with homogeneous heterogeneous reactions in the presence of carbon nanotubes.

Given the foregoing, it is witnessed that abundant studies are accessible in the literature focusing on non-Newtonian nanofluid flows over rotating disks. This geometry even becomes narrower if we talk about the flow of non-Newtonian nanofluid flow over a rotating disk with variable thickness in a permeable media with homogeneous-heterogeneous chemical reactions. But here in this study, the novelty is manifold including the surface catalyzed chemical reaction in addition to the homogeneous-heterogeneous chemical reactions. Secondly, the consideration of variable thermal conductivity and the viscosity instead of the constant and the non-Newtonian fluid Ostwald-de-Wale blended with melting heat boundary condition. The problem is solved numerically employing the bvp4c MATLAB built-in function. The salient outcomes of the model are discussed via graphs and tables. A comparison with a previously published paper is also added and an excellent concurrence is achieved in this regard.

The goal of this research is to answer the subsequent pertinent questions:

i. How surface catalyzed parameter influence the concentration profile?
ii. What is the association of the power-law index and variable viscosity?
iii. Which is more influential on the thickness of the boundary layer either the power-law index or variable thickness index?
iv. How do velocity and thermal profiles are influenced by disk thickness index?
v. How does the Schmidt number affect the concentration profile of chemical species for pseudoplastic fluid?
vi. How does the heat transfer process is affected by varying Prandtl number?

## Formulation of the problem

**Figure 1:**
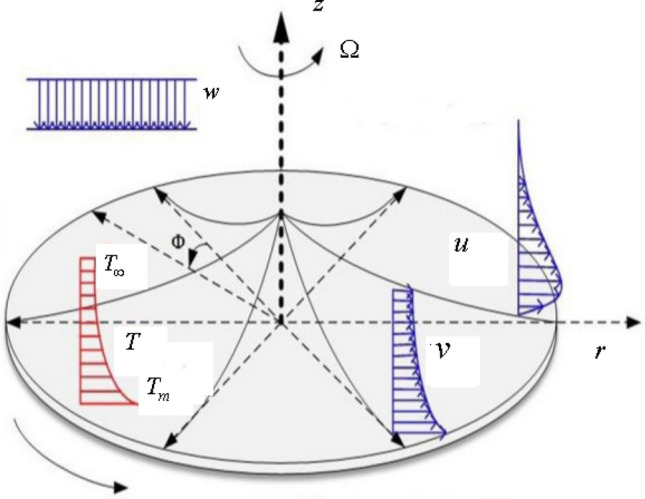
Geometrical sketch of the flow pattern.

The flow under consideration assumes the axisymmetric, laminar, steady flow of Ostwald-de Waele fluid in porous media driven by the rotation of disk of variable thickness with angular velocity $\Omega$ along z-direction with homogeneous–heterogeneous reactions. The heterogeneous reaction occurring on the surface of porous media is termed a surface catalyzed chemical reaction. Melting surface temperature is maintained at $T_{m}$ on the disk, while $T_{\infty }$ is representing the ambient fluid’s temperature (Fig. 1).

The homogeneous chemical reaction governed by the isothermal cubic autocatalytic reaction is written by the following chemical equation^45,46^

1 $$ A^{*} + 2B^{*} \to 3B^{*} , $$

While the heterogeneous reaction proceeds on the catalyst’s surface and fluid–solid interface and its order are one. The chemical equation for this reaction is given by

2 $$ A^{*} \to B^{*} , $$

here $A^{*}$ and $B^{*}$ are representing chemical species involved in a chemical reaction.

If we consider the concentration of the chemical species $A^{*}$ and $B^{*}$ is $a$ and $b$ respectively, then the rate of homogeneous reaction can be determined by using the following equation^40^

3 $$ \frac{\partial a}{{\partial t}} = \frac{\partial b}{{\partial t}} = - k_{c} ab^{2} , $$

The rate at which surface catalyzed chemical reaction proceeds can be estimated by the following equation^48^

4 $$ D_{A} \frac{\partial a}{{\partial n}} = - D_{B} \frac{\partial b}{{\partial n}} = - k_{s} a, $$

here $A^{*}$ and $B^{*}$ have the diffusion coefficient given by $D_{{A^{*} }}$ and $D_{{B^{*} }}$ respectively. $n$ is the normal vector of unit magnitude in the fluid direction.

The rate of reaction occurring in porous media is given by^48^

5 $$ r_{p} = - Sk_{s} a, $$

where the porous media has the interfacial surface area $S$.

The flow geometry is analyzed by considering the cylindrical coordinates $(r,\varphi ,z)$ under the assumption of $\frac{\partial p}{{\partial r}} = 0$ and $\frac{\partial p}{{\partial z}} = 0$. The governing equation with applied boundary conditions are^25,26^:

6 $$ \frac{u}{r} + \frac{\partial u}{{\partial r}} + \frac{\partial w}{{\partial z}} = 0, $$

7 $$ \rho \left( {u\frac{\partial u}{{\partial r}} - \frac{{v^{2} }}{r} + w\frac{\partial u}{{\partial z}}} \right) = \frac{\partial }{\partial z}\left( {\mu \frac{\partial u}{{\partial z}}} \right) - \frac{\nu }{{k^{*} }}u, $$

8 $$ \rho \left( {u\frac{\partial v}{{\partial r}} + \frac{uv}{r} + w\frac{\partial v}{{\partial z}}} \right) = \frac{\partial }{\partial z}\left( {\mu \frac{\partial v}{{\partial z}}} \right) - \frac{\nu }{{k^{*} }}v, $$

9 $$ \rho C_{p} \left( {u\frac{\partial T}{{\partial r}} + w\frac{\partial T}{{\partial z}}} \right) = \frac{\partial }{\partial z}\left( {\mu \frac{\partial T}{{\partial z}}} \right) + \tau \left[ {D_{B} \frac{\partial b}{{\partial z}}\frac{\partial T}{{\partial z}} + \frac{{D_{T} }}{{T_{\infty } }}\left( {\frac{\partial T}{{\partial z}}} \right)^{2} } \right], $$

10 $$ u\frac{\partial a}{{\partial r}} + w\frac{\partial a}{{\partial z}} = D_{A} \left( {\frac{{\partial^{2} a}}{{\partial z^{2} }}} \right) - k_{1} ab^{2} + \frac{{D_{T} }}{{T_{\infty } }}\left( {\frac{{\partial^{2} T}}{{\partial z^{2} }}} \right) - Sk_{2} a, $$

11 $$ u\frac{\partial b}{{\partial r}} + w\frac{\partial b}{{\partial z}} = D_{B} \left( {\frac{{\partial^{2} b}}{{\partial z^{2} }}} \right) + k_{1} ab^{2} + \frac{{D_{T} }}{{T_{\infty } }}\left( {\frac{{\partial^{2} T}}{{\partial z^{2} }}} \right) + Sk_{2} a, $$

With boundary conditions are^44^

12 $$ u = 0,\;v = r\Omega ,\;T = T_{m} ,\;D_{A} \frac{\partial a}{{\partial z}} = - D_{B} \frac{\partial b}{{\partial z}} = k_{2} a,\;{\text{at}}\;z = a\left( {\frac{r}{{R_{0} }} + 1} \right)^{ - m} , $$

13 $$ u = 0,\;v = 0,\;T = T_{\infty } ,\;a \to a_{o} ,\;b \to 0,\;{\text{as}}\;z \to \infty , $$

and

14 $$ k\left( {\frac{\partial T}{{\partial z}}} \right)_{z = 0} = \rho \left( {c_{s} \left( {T_{m} - T_{0} } \right) + \lambda^{*} } \right)w(r,0), $$

here $c_{s}$ denoting the solid surface heat capacity. $\lambda$ is the latent heat of the fluid. $T_{0}$ is the surface temperature and $T_{m}$ is the melting temperature. $R_{0}$ represents the feature radius. $a$ is the thickness coefficient of the disk.

The viscosity and thermal conductivity is defined^27^ as $\mu = \mu_{o} \left\{ {\left( {\frac{\partial u}{{\partial z}}} \right)^{2} + \left( {\frac{\partial v}{{\partial z}}} \right)^{2} } \right\}^{{\frac{n - 1}{2}}}$ and thermal conductivity^36^ $k = k_{o} \left\{ {\left( {\frac{\partial u}{{\partial z}}} \right)^{2} + \left( {\frac{\partial v}{{\partial z}}} \right)^{2} } \right\}^{{\frac{n - 1}{2}}}$ respectively for Ostwald-de-Waele fluid, $\mu_{o}$ and $k_{o}$ are the viscous and thermal consistency coefficient respectively. $n$ is the power-law index. For $n = 1$, we have Newtonian fluid. $0 < n < 1$ corresponds to pseudo-plastic fluid while for dilatant fluid we have $n > 1$. We shall define the dimensionless radius by $r^{*} = r/R_{o}$.

Assume the following similarity transformation:

15 $$ \begin{aligned} u & = r^{*} R_{0} \Omega F(\eta ),\;v = r^{*} R_{0} \Omega G(\eta ),\;T = \left( {T_{w} - T_{\infty } } \right)\theta + T_{\infty } , \\ w & = R_{0} \Omega \left( {\frac{{\Omega^{2 - n} R_{o}^{2} \rho }}{{\mu_{o} }}} \right)^{{\frac{1}{n + 1}}} (1 + r)^{ - m} H(\eta ), \\ \eta & = \frac{z}{{R_{o} }}\left( {\frac{{\Omega^{2 - n} R_{o}^{2} \rho }}{{\mu_{o} }}} \right)^{{\frac{1}{n + 1}}} (1 + r)^{m} ,\;a = a_{\infty } \phi ,\;b = a_{\infty } l, \\ \end{aligned} $$

Consider deformations as:

16 $$ F = f(\eta - \alpha ) = f(\xi ),\;G = g(\eta - \alpha ) = g(\xi ), $$

17 $$ H = h(\eta - \alpha ) = h(\xi ),\;\Theta = \theta (\eta - \alpha ) = \theta (\xi ), $$

The transformed partial differential equation including continuity and momentum equations are

18 $$ 2f + m\left( {\xi + \alpha } \right)\varepsilon f^{\prime } + h^{\prime } = 0, $$

19 $$ f^{2} + m\left( {\xi + \alpha } \right)\varepsilon ff^{\prime } - g^{2} + f^{\prime } h = A\left( {Bf^{\prime } } \right)^{\prime } - Kf, $$

20 $$ 2fg + m\left( {\xi + \alpha } \right)\varepsilon fg^{\prime } + g^{\prime } h = A\left( {Bg^{\prime } } \right)^{\prime } - Kg, $$

21 $$ m\left( {\xi + \alpha } \right)\varepsilon f\theta^{\prime } + \theta^{\prime } h = \frac{1}{\Pr }A\left( {B\theta^{\prime } } \right)^{^{\prime}} + AB\left( {N_{b} \theta^{\prime } \phi^{\prime } + N_{t} \theta^{\prime 2} } \right), $$

22 $$ A\left( B \right)\left( {\frac{1}{Sc}\left( {\psi^{\prime \prime } + \frac{{N_{t} }}{{N_{b} }}\theta^{\prime \prime } } \right)} \right) - K_{1} \psi \phi^{2} + m\left( {\xi + \alpha } \right)\varepsilon f\psi^{\prime } + h\psi^{\prime } - k_{vs} \psi = 0, $$

23 $$ AB\left( {\frac{1}{\delta Sc}\left( {\phi^{\prime \prime } + \frac{{N_{t} }}{{N_{b} }}\theta^{\prime \prime } } \right)} \right) + K_{1} \psi \phi^{2} + m\left( {\xi + \alpha } \right)\varepsilon f\phi^{\prime } + h\phi^{\prime } + k_{vs} \psi = 0, $$

Assuming the diffusion coefficient of chemical species same we take $\psi + \phi = 1$ and $\delta = 1$, we obtain,

24 $$ \left( {\frac{AB}{{Sc}}\left( {\psi^{\prime \prime } + \frac{{N_{t} }}{{N_{b} }}\theta^{\prime \prime } } \right)} \right) - K_{1} \psi \left( {1 - \psi } \right)^{2} + m\left( {\xi + \alpha } \right)\varepsilon f\psi^{\prime } + h\psi^{\prime } - k_{vs} \psi = 0, $$

For simplicity we denote $A = r^{n - 1} (1 + r)^{m(n + 1)} ,$

$$ B = \left\{ {\left( {f^{\prime } } \right)^{2} + \left( {g^{\prime } } \right)^{2} } \right\}^{{\frac{n - 1}{2}}} , $$

The transformed boundary conditions take the following form

25 $$ \begin{aligned} f(0) & = 0,\;g(0) = 1,\;\theta (0) = 0,\;\left( {\text{Re}} \right)^{{\frac{1}{n + 1}}} \left( {1 + r} \right)^{m} \psi^{\prime } (0) = K_{2} \psi (0), \\ f(\infty ) & = 0,\;g(\infty ) = 0,\;\theta (\infty ) = 1,\;\psi (\infty ) = 1, \\ \end{aligned} $$

and

26 $$ \theta^{\prime } (0)r^{n - 1} (1 + r)^{m(n + 1)} \left\{ {\left( {f^{\prime } } \right)^{2} + \left( {g^{\prime } } \right)^{2} } \right\}^{{\frac{n - 1}{2}}} = \frac{\Pr }{{Ma}}h(0), $$

Presenting the following deformations

27 $$ F = f(\xi - \alpha ) = f(\eta ),\;G = g(\xi - \alpha ) = g(\eta ), $$

28 $$ H = h(\xi - \alpha ) = h(\eta ),\;\Theta = \theta (\xi - \alpha ) = \theta (\eta ), $$

where

29 $$ \begin{aligned} \alpha & = \left( {\frac{a}{{R_{o} }}} \right).\left( {\frac{{\Omega^{2 - n} R_{o}^{2} \rho }}{{\mu_{o} }}} \right)^{{\frac{ - 1}{{n + 1}}}} ,\;{\text{Re}} = \frac{{\Omega^{2 - n} R_{o}^{2} \rho }}{{\mu_{o} }},\delta = \frac{{D_{A} }}{{D_{B} }},\;K_{1} = \frac{{a_{\infty }^{2} k_{1} }}{\Omega },\;K_{2} = \frac{{k_{2} \sqrt \nu }}{{D_{A} \sqrt \Omega }} \\ S_{\nu } & = \frac{{SD_{A} }}{{\sqrt {\Omega \nu } }},\;k_{vs} = S_{\nu } K_{2} ,\;K = \frac{\nu }{{\Omega k^{*} }},\;Ma = \frac{{\lambda^{*} + c_{s} \left( {T_{m} - T_{o} } \right)}}{{\left( {T_{m} - T_{\infty } } \right)C_{p} }},\;\Pr = \frac{{\mu_{o} C_{p} }}{{\lambda_{o} }} \\ \end{aligned} $$

here $\alpha$ is the dimensionless thickness coefficient of the disk, ${\text{Re}}$ is the Reynolds number, $\delta$ is the quotient of diffusion coefficients,$K_{1}$ and $K_{2}$ are the measurement of the strength of homogeneous and heterogeneous reaction respectively. $S_{\nu }$ parameter of interfacial area, $K,k_{vs}$ and $Ma$ are the porosity, surface catalyzed parameter, and melting heat parameter. $\Pr$ is the Prandtl number.

Moreover, radial and tangential shear stress can be estimated by the following equation

30 $$ \tau_{{w_{r} }} = \mu \left. {\frac{\partial u}{{\partial z}}} \right|_{{z = A\left( {\left( {\frac{r}{{R_{o} }}} \right) + 1} \right)^{ - m} }} = \mu_{o} \left\{ {\left( {\frac{\partial u}{{\partial z}}} \right)^{2} + \left( {\frac{\partial v}{{\partial z}}} \right)^{2} } \right\}^{{\frac{n - 1}{2}}} \left. {\frac{\partial u}{{\partial z}}} \right|_{{z = A\left( {\left( {\frac{r}{{R_{o} }}} \right) + 1} \right)^{ - m} }} , $$

31 $$ \tau_{{w_{\varphi } }} = \mu \left. {\frac{\partial v}{{\partial z}}} \right|_{{z = A\left( {\left( {\frac{r}{{R_{o} }}} \right) + 1} \right)^{ - m} }} = \mu_{o} \left\{ {\left( {\frac{\partial u}{{\partial z}}} \right)^{2} + \left( {\frac{\partial v}{{\partial z}}} \right)^{2} } \right\}^{{\frac{n - 1}{2}}} \left. {\frac{\partial v}{{\partial z}}} \right|_{{z = A\left( {\left( {\frac{r}{{R_{o} }}} \right) + 1} \right)^{ - m} }} , $$

Heat flux is defined as

32 $$ q_{w} = - \lambda \left. {\frac{\partial T}{{\partial z}}} \right|_{{z = A\left( {\left( {\frac{r}{{R_{o} }}} \right) + 1} \right)^{ - m} }} = \lambda_{o} \left\{ {\left( {\frac{\partial u}{{\partial z}}} \right)^{2} + \left( {\frac{\partial v}{{\partial z}}} \right)^{2} } \right\}^{{\frac{n - 1}{2}}} \left. {\frac{\partial T}{{\partial z}}} \right|_{{z = A\left( {\left( {\frac{r}{{R_{o} }}} \right) + 1} \right)^{ - m} }} , $$

## Numerical scheme

The system of Eqs. (18)–(24) is transformed to a system of first-order differential equations and solved using the MATLAB software function bvp4c. To get a numerical solution, a tolerance of 10^–6^ is set for the initial approximations. This presumed prior guess must satisfy Eq. (25). The transformed coupled non-linear ordinary differential equations are computed using the bvp4c technique. To get the system of first-order equations, new variables are first introduced:

33 $$ \begin{aligned} f & = Y_{1} ,\;f^{\prime } = Y_{2} ,\;f^{\prime \prime } = yy_{1} ,\;g = Y_{3} ,\;g^{\prime } = Y_{4} ,\;g^{\prime \prime } = yy_{2} ,\;h = Y_{5} ,\;h^{\prime } = yy_{3} ,\;\theta = Y_{6} , \\ \theta^{\prime } & = Y_{7} ,\;\theta^{\prime \prime } = yy_{4} ,\;\psi = Y_{8} ,\;\psi^{\prime } = Y_{9} ,\;\psi^{\prime \prime } = yy_{5} , \\ \end{aligned} $$

On applying the transformation we obtained the following system of differential equations as.

34 $$ yy_{1} = \frac{\begin{gathered} A\left\{ {B + C(n - 1)Y_{3}^{2} } \right\} \times (Y_{1}^{2} + m(\xi + \alpha )\varepsilon Y_{1} Y_{2} - Y_{3}^{2} + Y_{2} Y_{5} ) \hfill \\ - AC(n - 1)f^{\prime } g^{\prime } (2Y_{1} Y_{3} + m(\xi + \alpha )\varepsilon Y_{1} Y_{3} + Y_{4} Y_{5} ) \hfill \\ \end{gathered} }{\begin{gathered} A\left\{ {B + C(n - 1)Y_{2}^{2} } \right\} \times A\left\{ {B + C(n - 1)Y_{3}^{2} } \right\} + \hfill \\ A^{2} C^{2} (n - 1)^{2} g^{\prime 2} f^{\prime } f \hfill \\ \end{gathered} }, $$

35 $$ yy_{2} = \frac{{2Y_{1} Y_{3} + m(\xi + \alpha )\varepsilon Y_{1} Y_{3} + Y_{4} Y_{5} - AC(n - 1)fg^{\prime}yy_{1} }}{{A\left\{ {B + C(n - 1)Y_{3}^{2} } \right\}}}, $$

36 $$ yy_{3} = - 2Y_{1} - m(\xi + \alpha )\varepsilon Y_{2} , $$

37 $$ yy_{4} = \frac{\begin{gathered} \Pr \times m(\xi + \alpha )\varepsilon Y_{1} Y_{7} + Y_{7} Y_{5} - A \times B \times \hfill \\ \Pr (Nb \times Y_{7} \times Y_{9} + Nt \times Y_{7}^{2} ) - A \times C(n - 1)Y_{7} \hfill \\ \end{gathered} }{AB}, $$

38 $$ yy_{5} = \frac{{\frac{Nt}{{Nb}}yy_{3} \times A \times B - Sc(m\eta \varepsilon Y_{1} Y_{9} + Y_{5} Y_{9} ) + ScK_{1} Y_{8} (1 - Y_{8} )^{2} - k_{vs} Y_{8} }}{AB}, $$

39 $$ \begin{aligned} & Y_{1} (0) = 0,\;Y_{3} (0) = 1,\;Y_{6} (0) = 1,\;Y_{7} (0)AB = \frac{\Pr }{{Ma}}Y_{5} (0), \\ & \left( {\text{Re}} \right)^{{\frac{1}{n + 1}}} \left( {1 + r} \right)^{m} Y_{9} (0) = K_{2} Y_{8} (0),\;Y_{1} (\infty ) = 0,\;Y_{3} (\infty ) = 0,\;Y_{6} (\infty ),\;Y_{8} (\infty ) = 1 \\ \end{aligned} $$

For simplicity, we use the following symbols

40 $$ A = r^{n - 1} (1 + r)^{m(n + 1)} ,\;B = \left\{ {\left( {f^{\prime } } \right)^{2} + \left( {g^{\prime } } \right)^{2} } \right\}^{{\frac{n - 1}{2}}} ,\;C = \left\{ {\left( {f^{\prime } } \right)^{2} + \left( {g^{\prime } } \right)^{2} } \right\}^{{\frac{n - 3}{2}}} , $$

## Results and discussion

**Figure 2:**
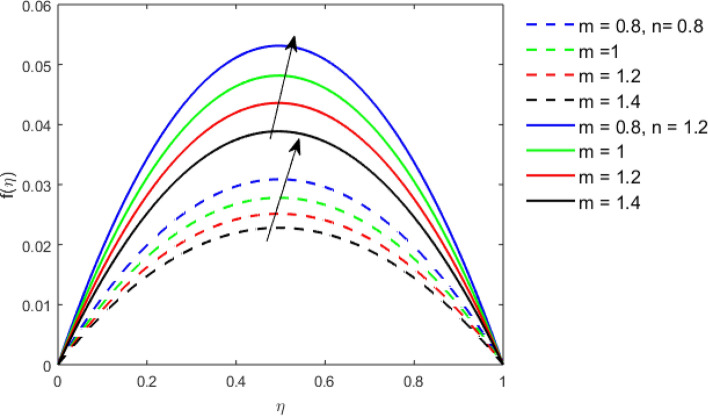
The Radial velocity profile for decreasing power-law fluid index with thickness index $m = 0.8,r = 0.6,\Pr = 1$ of a disk.

**Figure 3:**
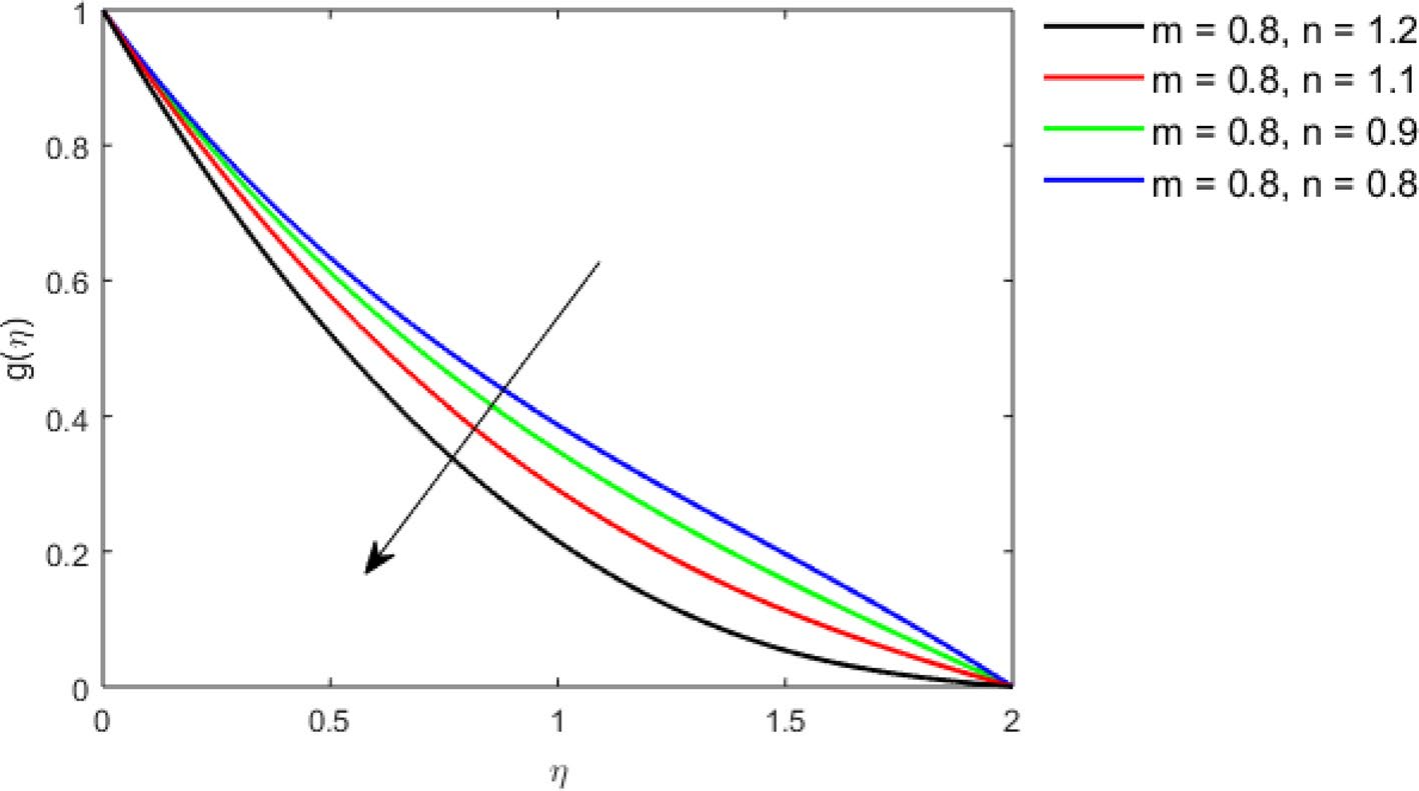
The Tangential velocity profile for decreasing power-law fluid index with thickness index $m = 0.8,r = 0.6,\Pr = 1$ of a disk.

**Figure 4:**
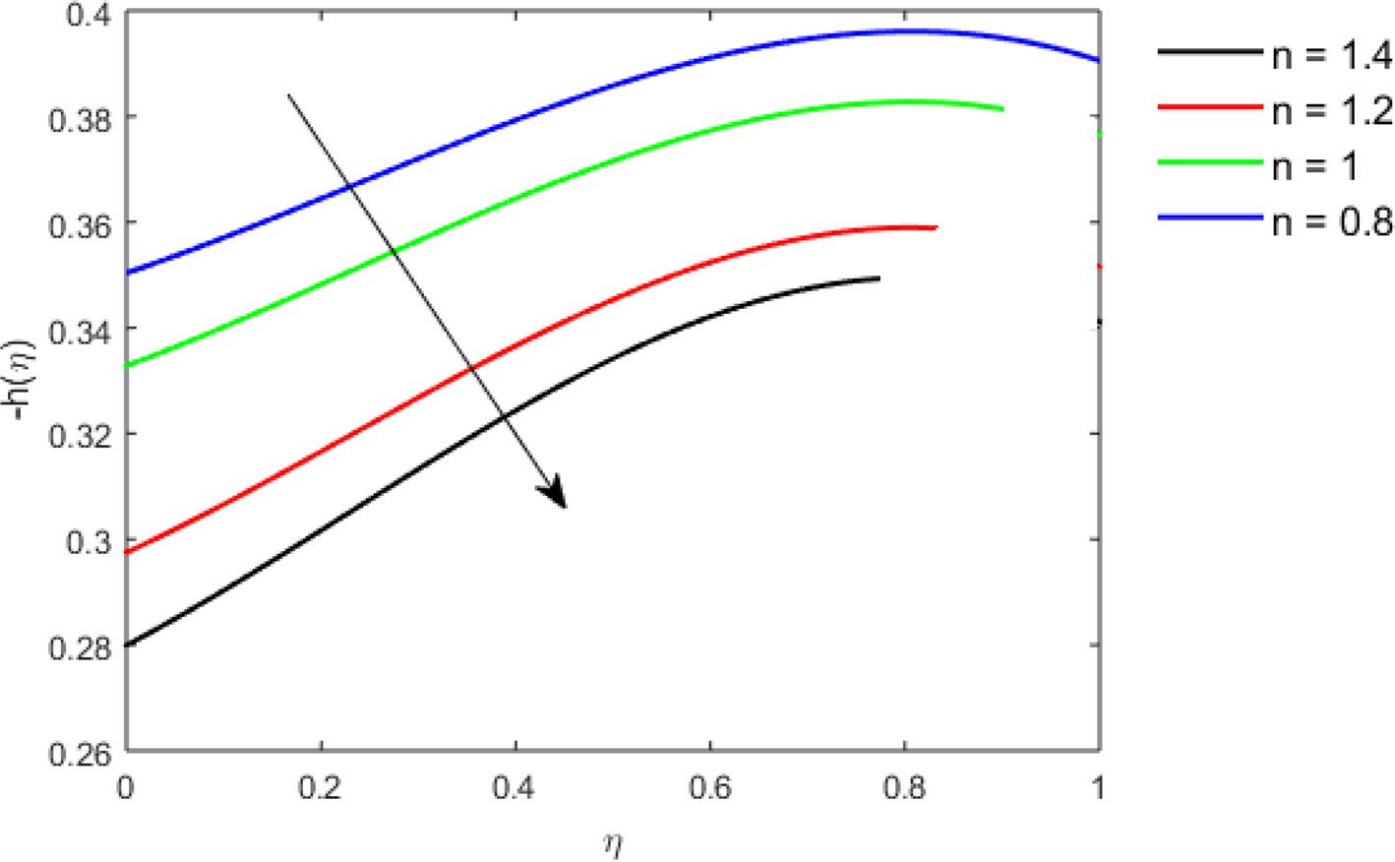
The Axial velocity profile for decreasing power-law fluid index with thickness index of disk $m = 0.8,r = 0.6,\Pr = 1$.

**Figure 5:**
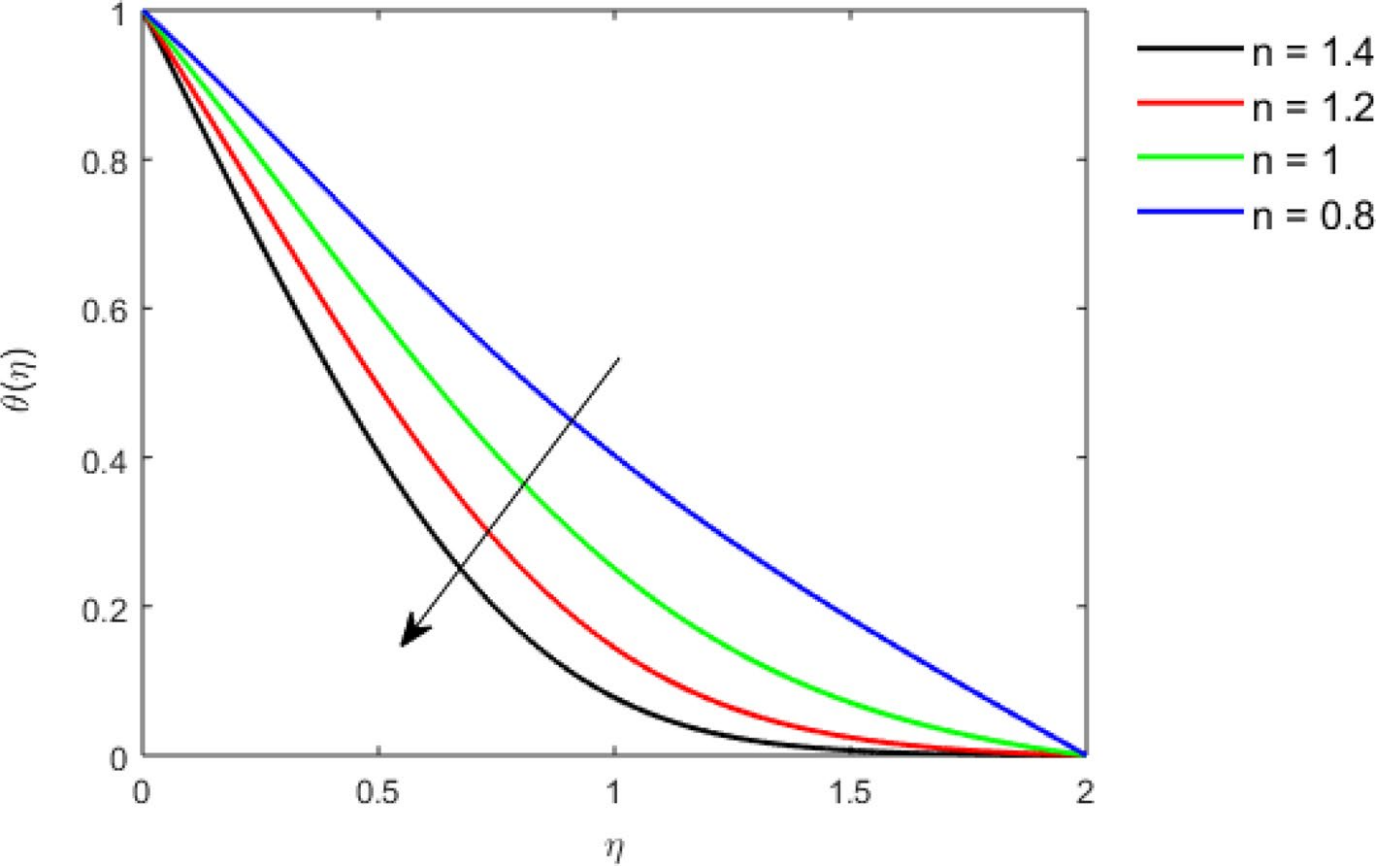
Thermal profile for decreasing power-law fluid index with thickness index $m = 0.8,r = 0.6,\Pr = 1$ of a disk.

**Figure 6:**
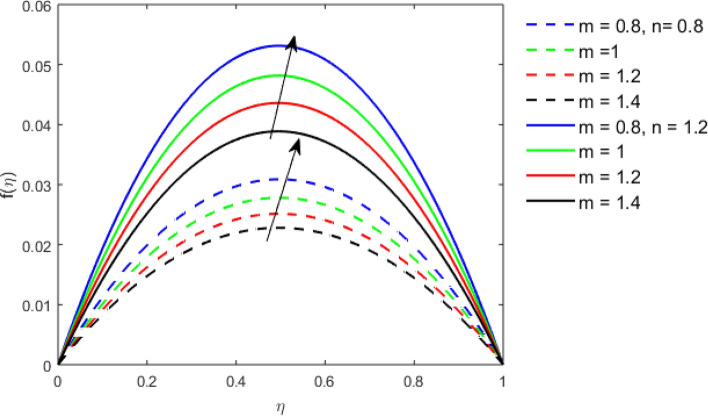
The Radial velocity profile for increasing thickness index $m$ of disk for Pseudoplastic and dilatant fluid.

**Figure 7:**
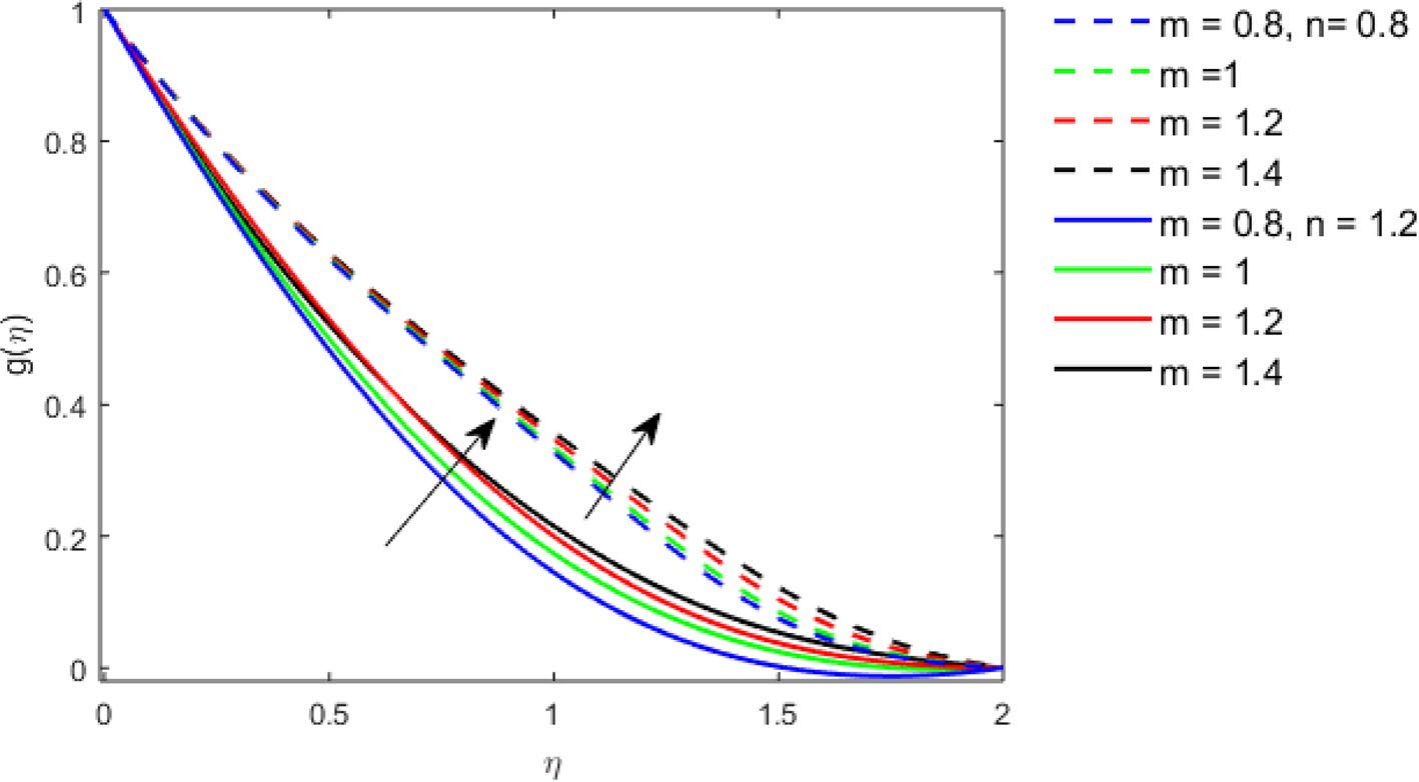
The Tangential velocity profile for increasing thickness index $m$ of disk for Pseudoplastic and dilatant fluid.

**Figure 8:**
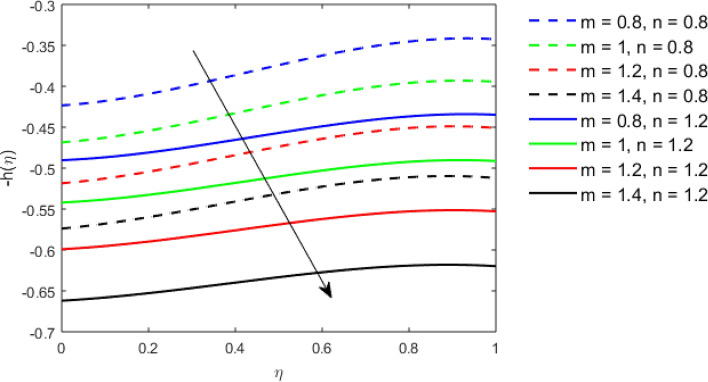
The Axial velocity profile for increasing thickness index $m$ of disk for Pseudoplastic and dilatant fluid.

**Figure 9:**
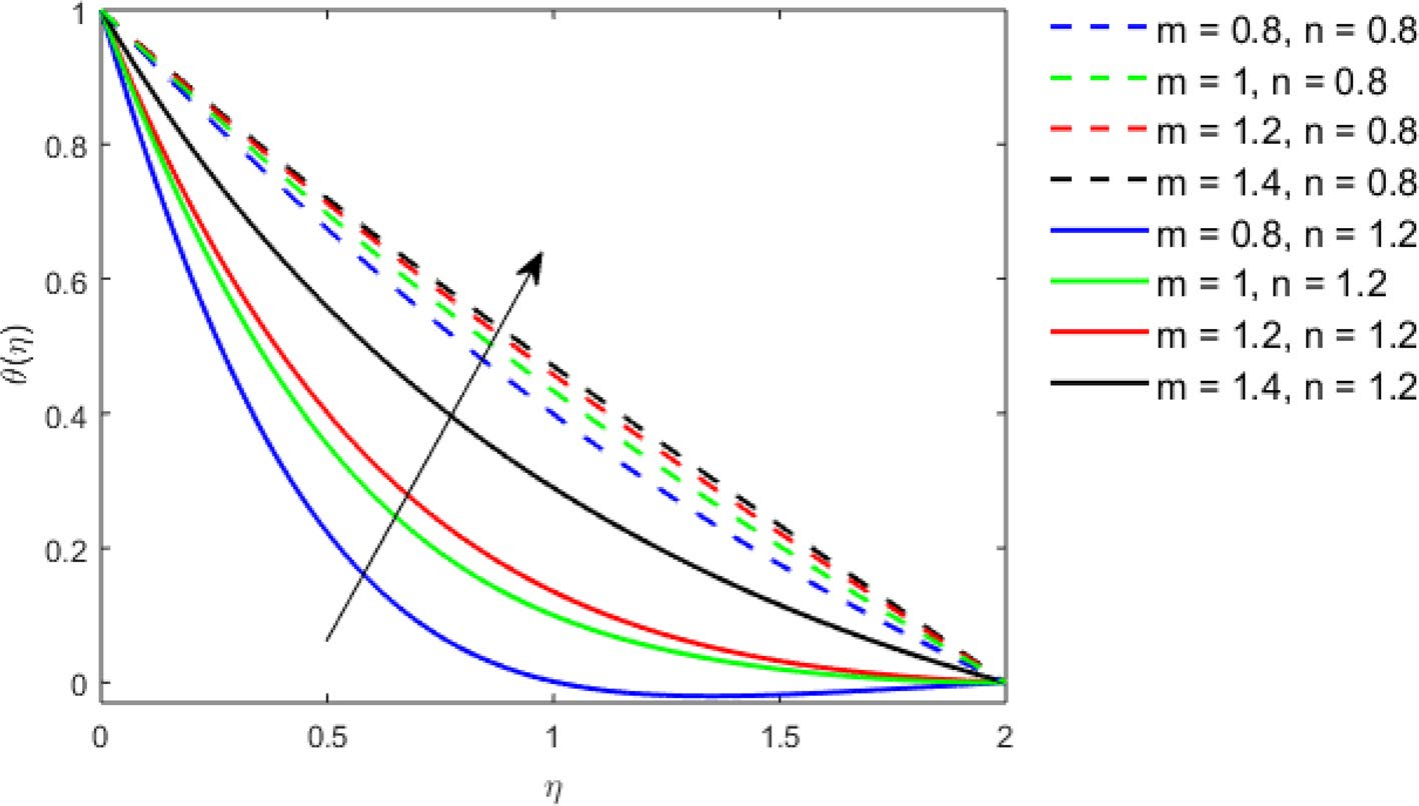
Thermal profile for increasing thickness index $m$ of disk for Pseudoplastic and dilatant fluid.

**Figure 10:**
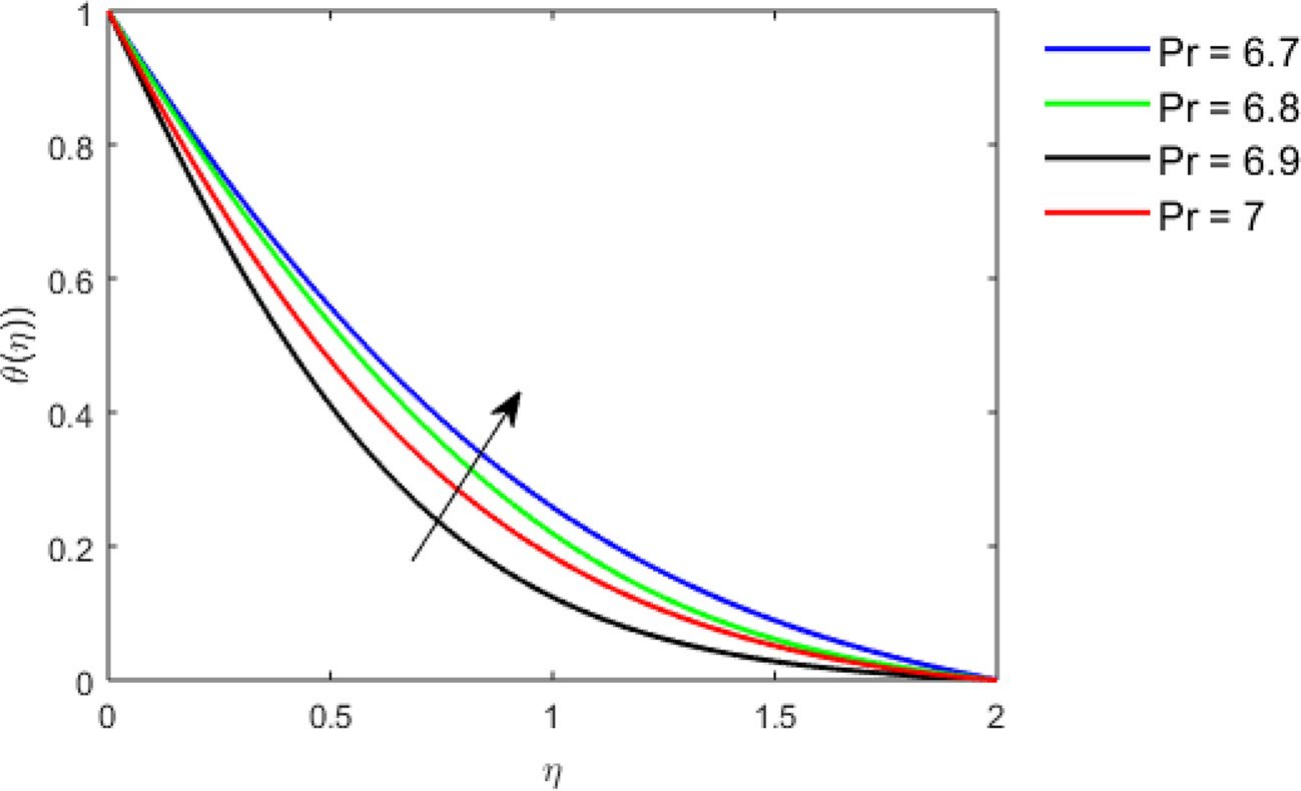
Thermal profile for increasing $\Pr$, with $n = 1.1,m = 2,r = 0.6$.

**Figure 11:**
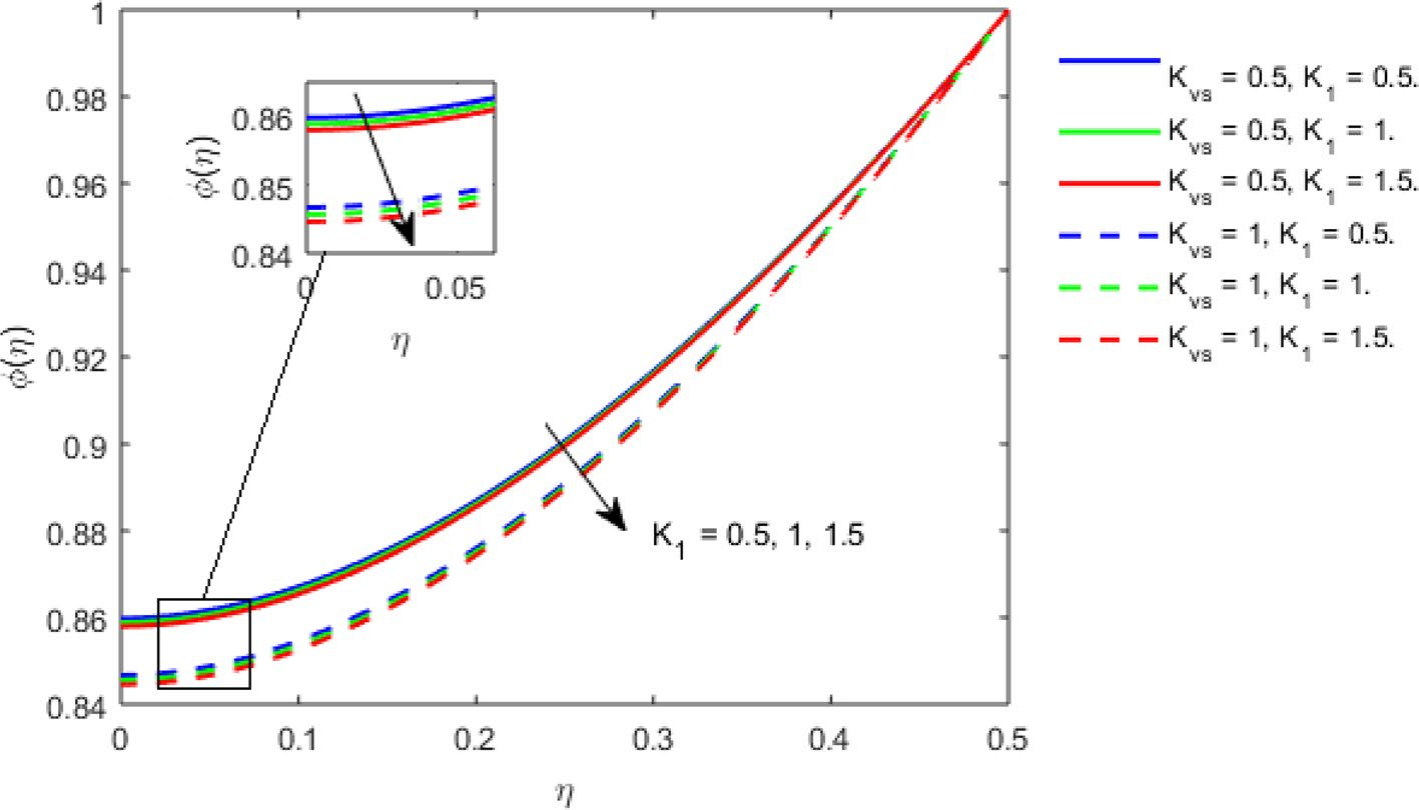
Concentration profile for increasing $K_{1} ,$ and $K_{vs} ,$ for Dilatant fluid by keeping thickness index $m = 0.8$.

**Figure 12:**
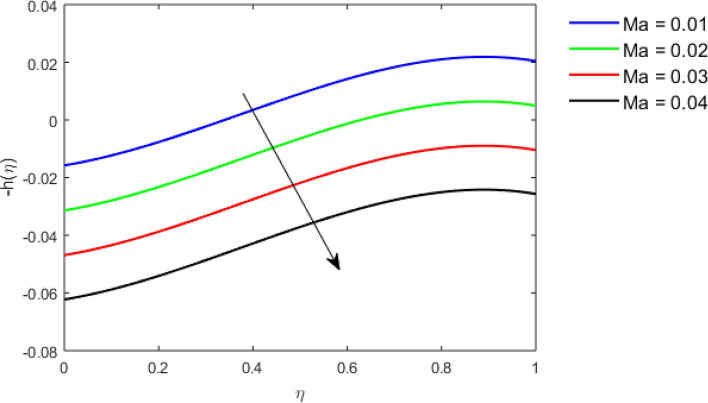
The Axial velocity profile for melting heat parameter $Ma,$ for Pseudoplastic fluid.

**Figure 13:**
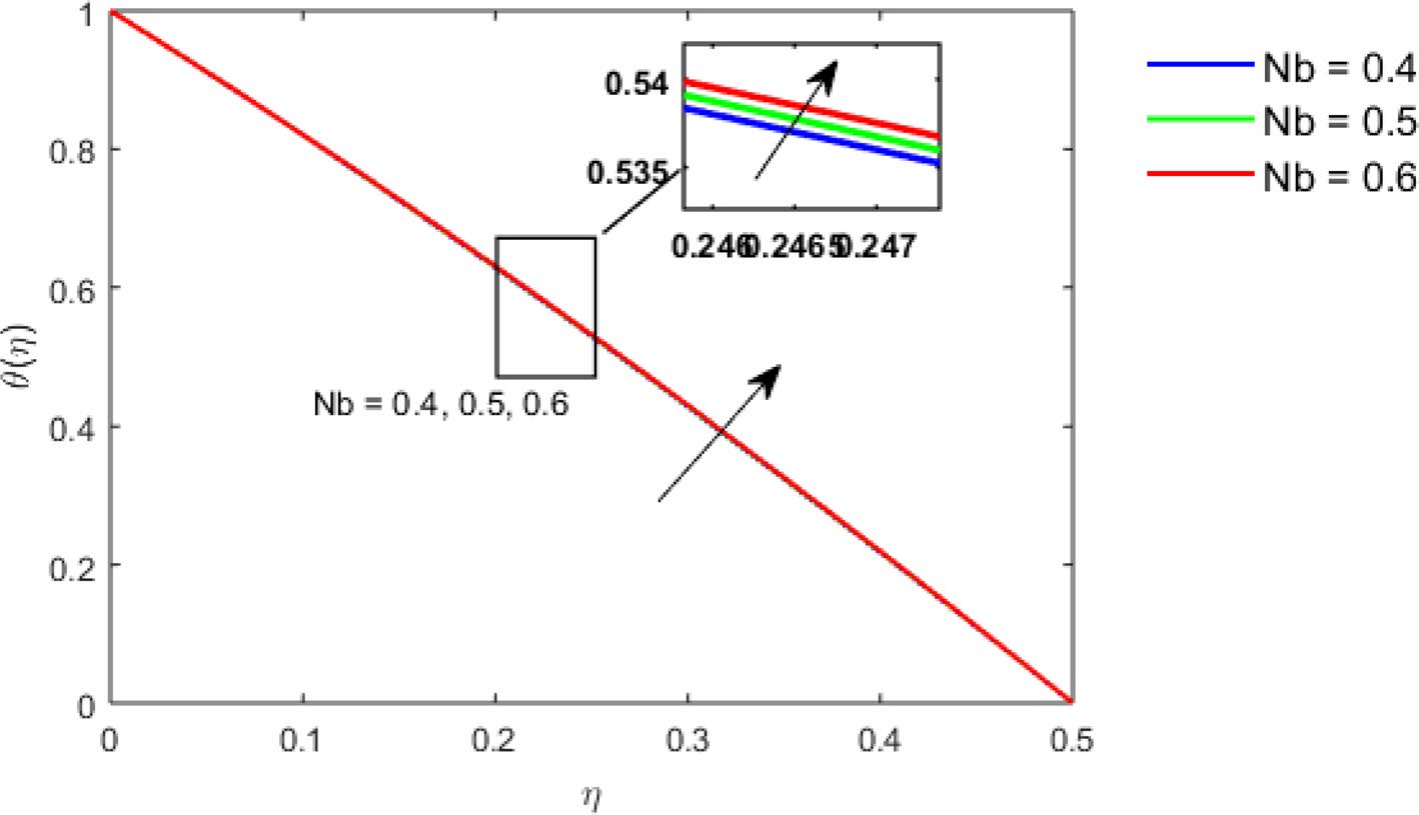
Thermal profile for increasing $Nb,$ for Pseudoplastic fluid by keeping $m = 0.8$.

**Figure 14:**
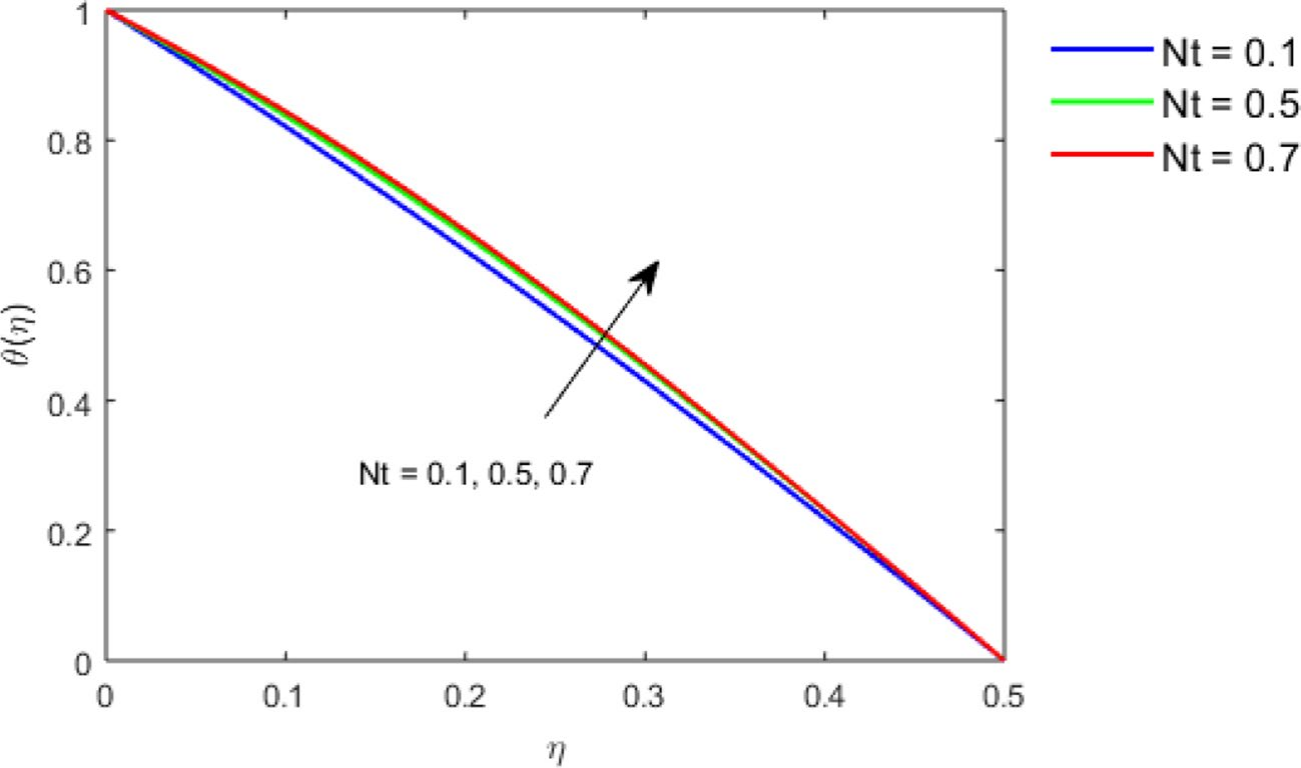
Thermal profile for increasing $Nt,$ for Pseudoplastic fluid by keeping $m = 0.8$.

**Figure 15:**
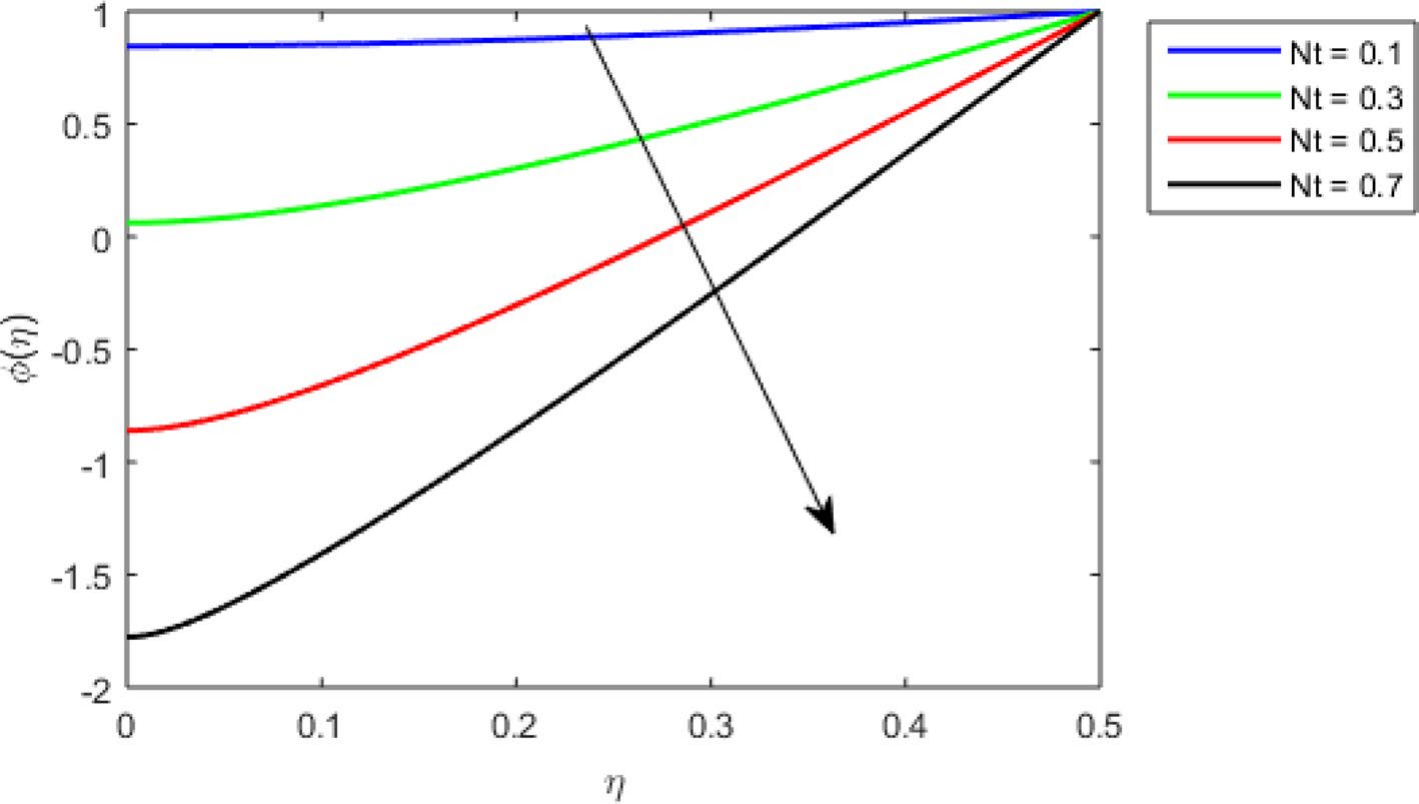
Thermal profile for increasing $Nt,$ for Pseudoplastic fluid by keeping $m = 0.8$.

**Figure 16:**
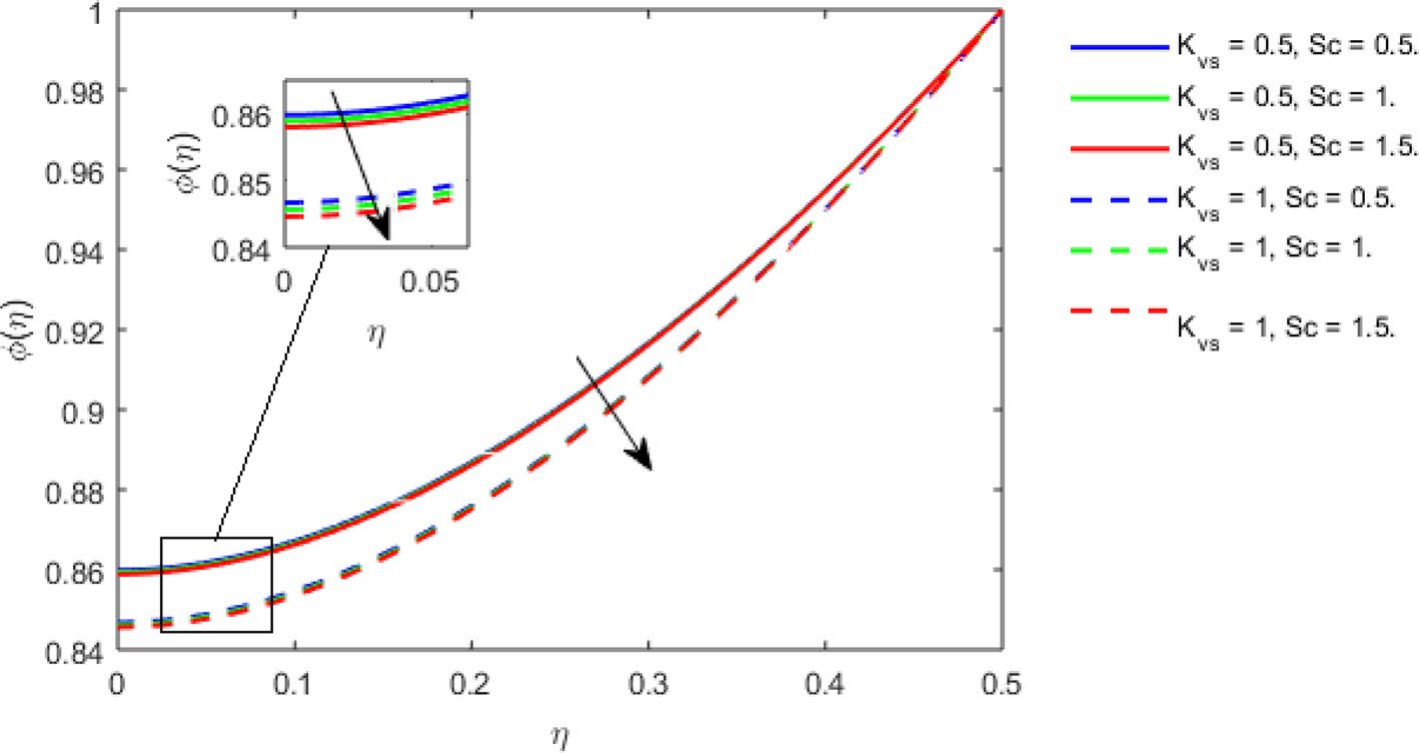
Concentration profile for increasing $Sc,K_{vs} ,$ for Dilatant fluid by keeping thickness index $m = 0.8$.

**Figure 17:**
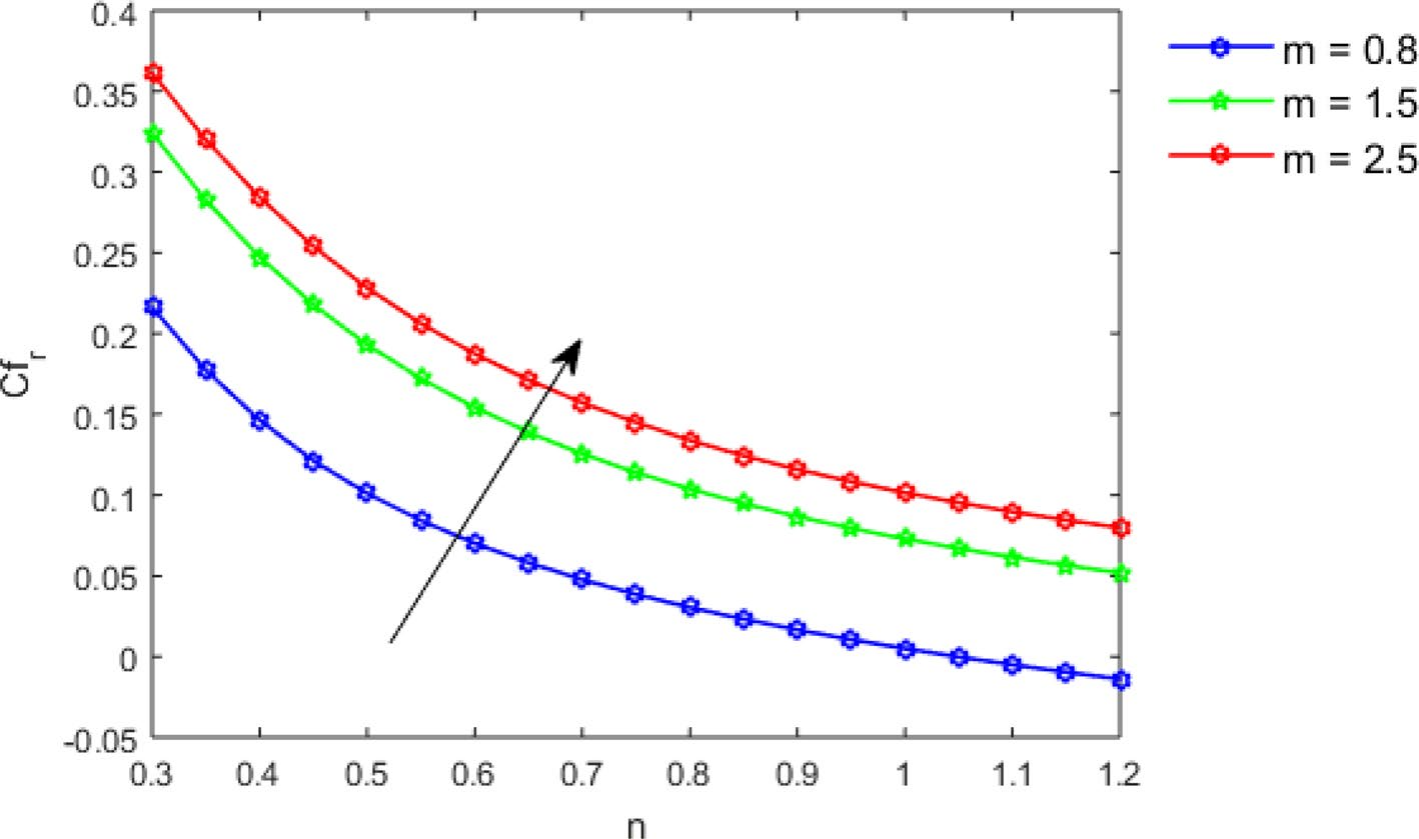
Coefficient of drag force in a radial direction by increasing $m,$ for $n < 1,n = 1$ and $n > 1$.

**Figure 18:**
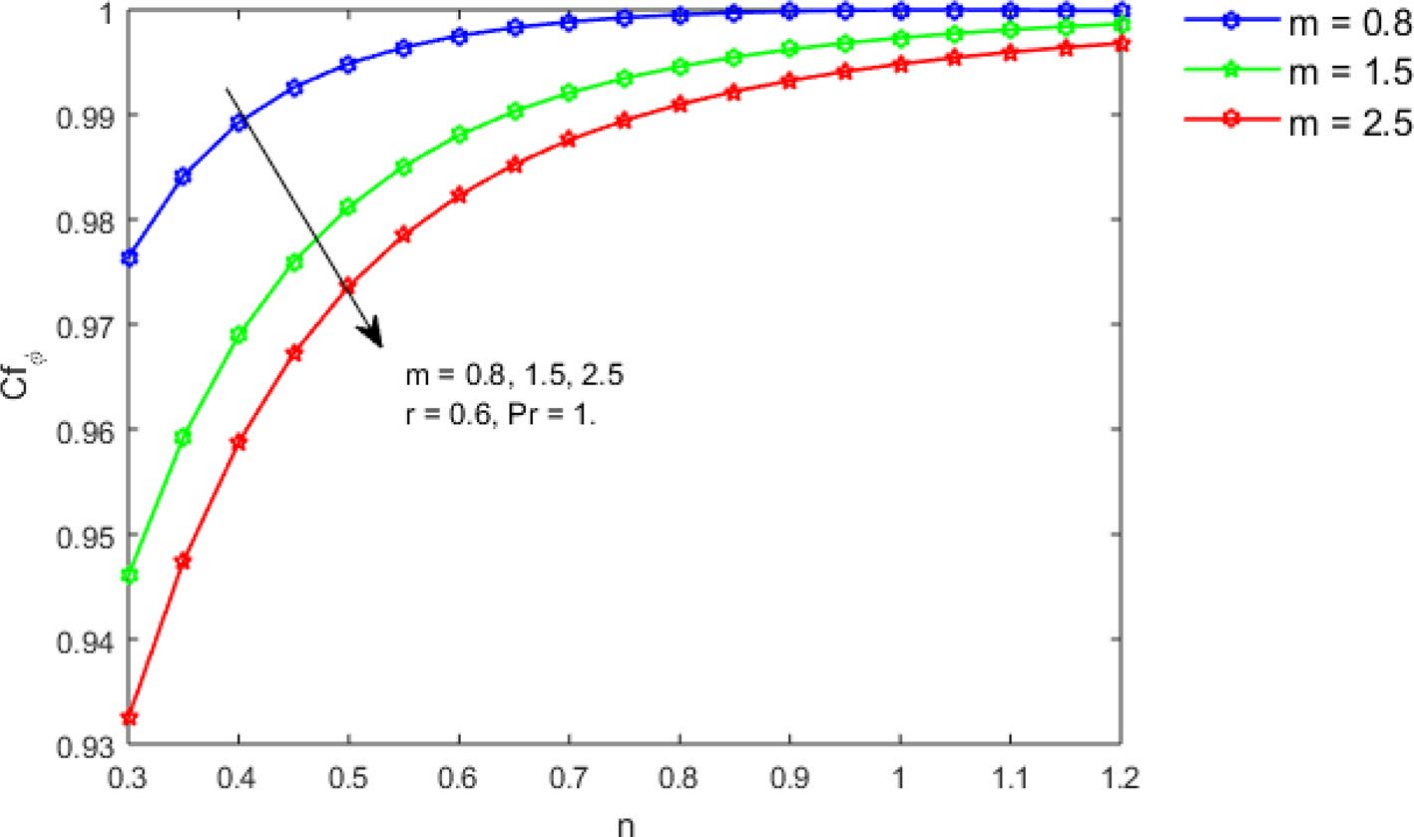
Coefficient of drag force in tangential direction by increasing $m$, for $n < 1,n = 1$ and $n > 1$.

**Figure 19:**
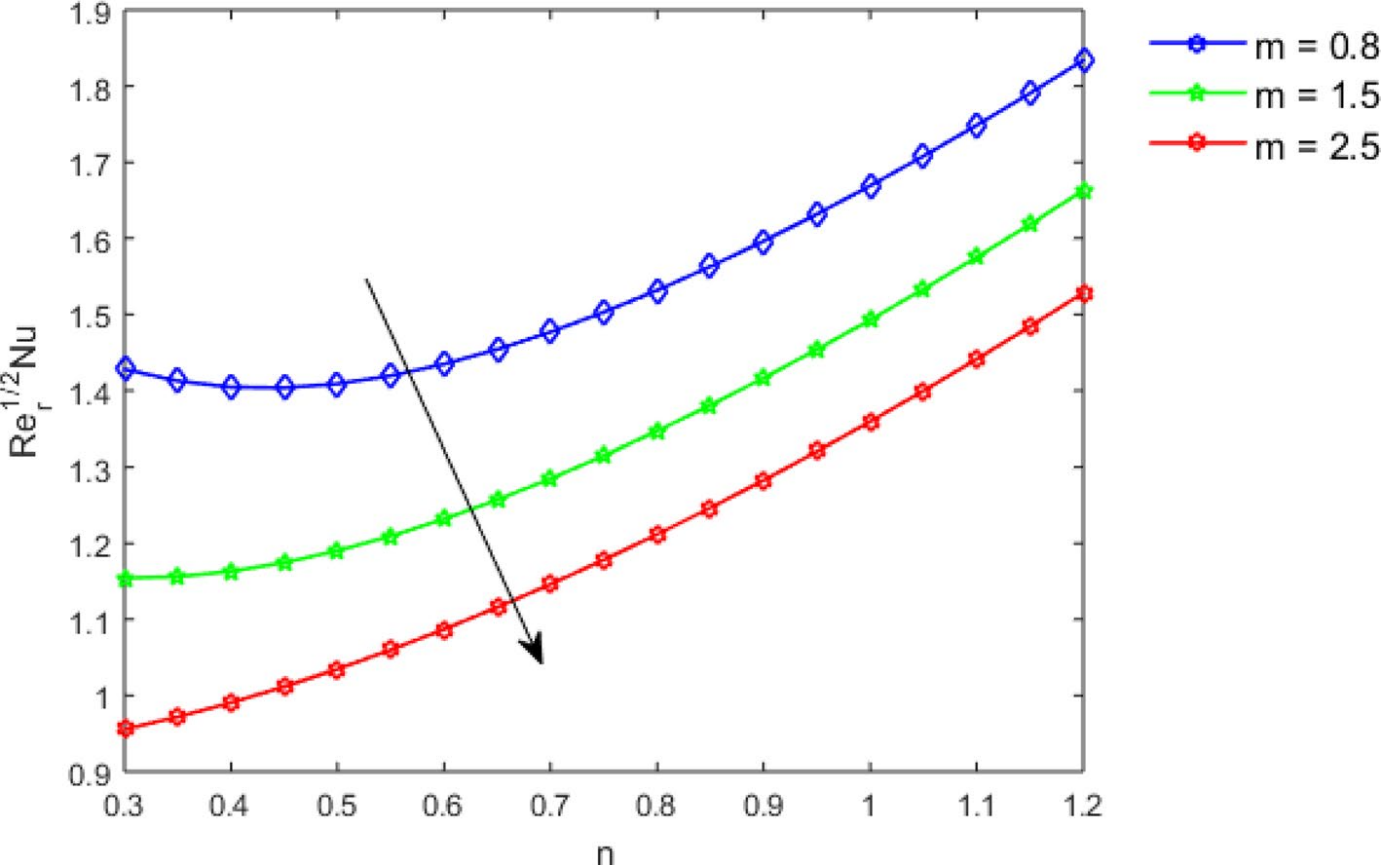
Heat transfer rate by increasing $m$, for $n < 1,n = 1$ and $n > 1$,$r = 0.6,\Pr = 1.$

**Figure 20:**
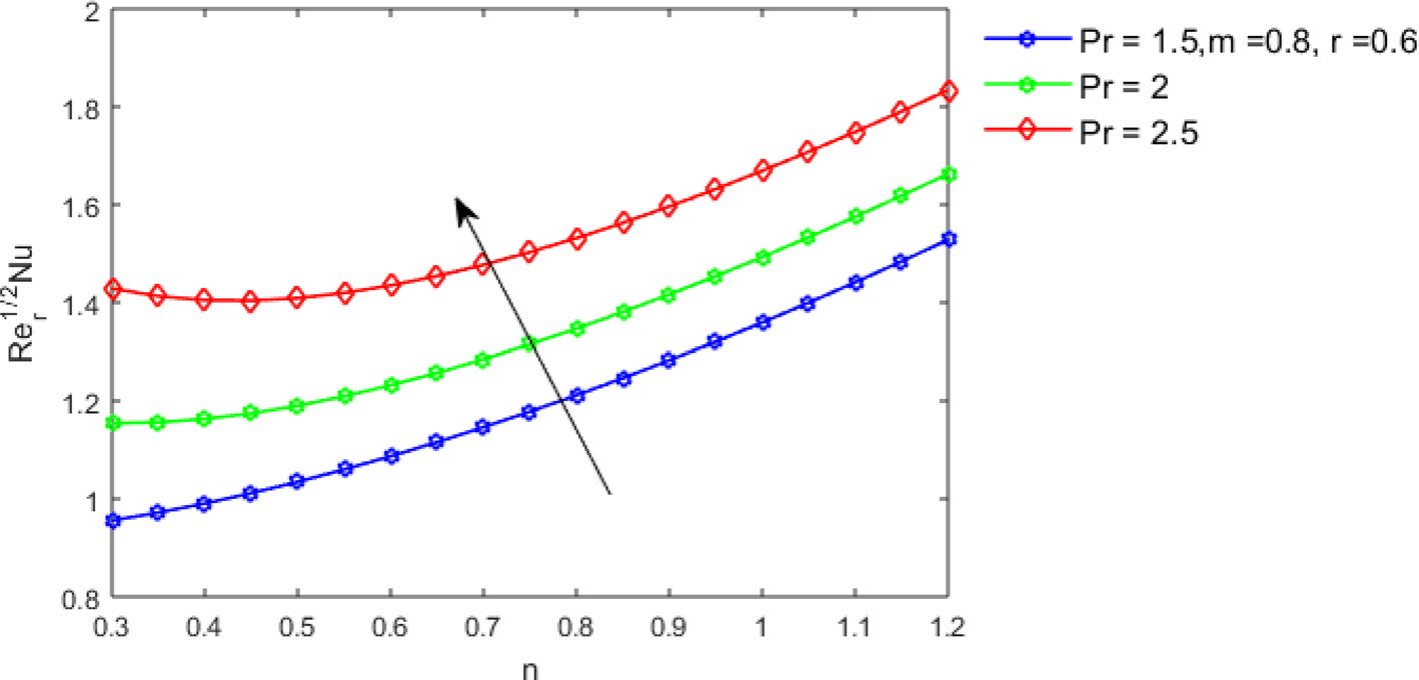
Heat transfer rate by increasing $\Pr ,$ for $n < 1,n = 1$ and $n > 1$.

**Figure 21:**
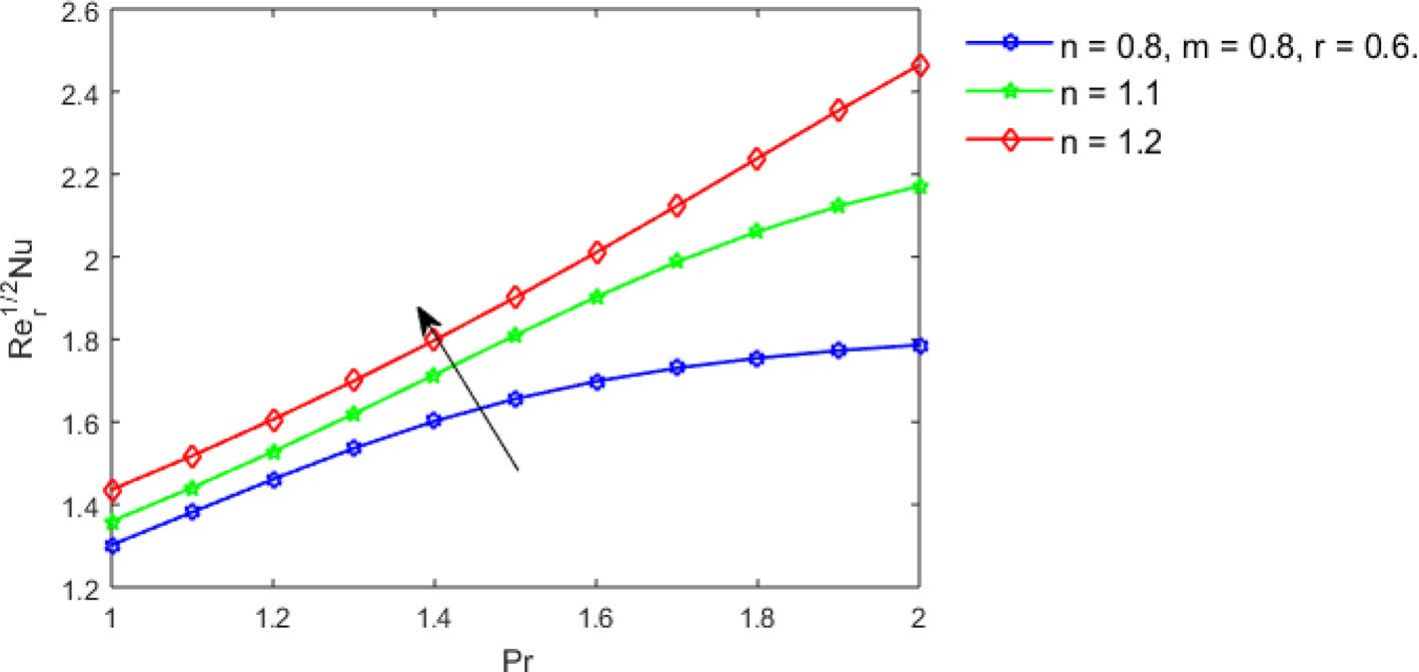
Heat transfer rate by the power-law index $n$ for increasing $\Pr$.

This section studies the axial, radial tangential, thermal, and concentration profile for arising pertinent parameters with disk thickness index and power-law index. Figure 2 represents the radial velocity profile for increasing power-law index by keeping the thickness index of the disk constant. Non-Newtonian rheology modestly affects the radial velocity profile for small values of $\eta$. For pseudo-plastic fluids, the peak values are attained for the radial velocity profile. Increasing pseudo-plasticity indicates that the accuracy of boundary layer approximations deteriorates. In fact, increasing the power-law index leads to monotonic thickening of boundary layers. Rotating disks exert the centrifugal force in the outward direction causing the outward radial flow and inward axial flow. Moreover, peak values tend to reduce as we check for dilatant fluids. Figure 3 is the depiction of decreasing shear-driven motion by fixing the thickness index of the disk $m = 0.8$ and increasing the power-law index. The non-Newtonian rheology is exhibited by varying power-law index which causes the fluids parallel to the disk to perforate otherwise stagnant fluid. Figure 4 represents the graphical behavior axial velocity profile for increasing power-law index to study the non-Newtonian rheology. The decline in axial velocity is due to the centrifugal force causing the axial inflow. The viscosity function is dependent upon viscous consistency coefficient. Increasing power-law index results in the increase of variable viscosity, which in turn responsible for the thickening of the boundary layer. And hence the significant enhancement of the flow occurs in the axial direction to compensate the radial outflow. Figure 5 exhibits the decrease of the thermal profile from shear-thinning fluid to shear-thickening fluid. Increasing the power-law index by fixing disk thickness $m = 0.8$ will cause the reduction in thermal boundary layer thickness. Which causes the upsurge in heat transfer efficiency. Figure 6 is drawn for radial velocity profile of dilatant and pseudoplastic fluid by varying thickness of the disk. The graphical analysis of non-Newtonian rheology reveals that the power-law index is more influential than the thickness index of the disk. In the regime of the disk, the radial velocity upshoot occurs increasing the thickness index of disk. In the boundary layer region, the shear stress is firstly positive and increasing and reaches to its maximum values and then it falls, causing the radial velocity profile to go to zero. Increasing the thickness index of disk, the boundary layer gets thicker. For pseudo-plasticity, the peak value is small as compared to the dilatant fluids. Figure 7 is drawn for tangential velocity profile for pseudoplastic and dilatant fluids by varying index thickness of the disk. The tangential velocity profile escalates by increasing the thickness index of disk. Figure 8 represents the axial inflow by varying wall thickness parameter for dilatant and pseudoplastic fluids. The power-law index is more influential as compared to the wall thickness index. Figure 9 is the depiction of the thermal profile for varying thickness index of the disk. The trend for increasing disk thickness index is again increasing. The reason for up shooting in the tangential, axial, and thermal profile is that the velocity and thermal boundary layer thickness enhances for increasing disk index thickness. Figure 10 exhibits the thermal profile for escalating $\Pr$. The increase in thermal profile causes the increase in thermal conductivity which leads to more surface heat transfer rate. Resultantly thermal boundary layer thickness gets thicker. Figure 11 depicts the concentration profile for homogeneous reaction parameter and surface catalyzed parameter for shear thickening fluids. The enhancement in surface catalyzed and homogeneous reaction parameter causes the thinning of the concentration boundary layer for non-Newtonian rheology. The reactants consume during homogeneous reaction proceeds. By increasing the homogeneous reaction parameter, the reaction proceeds more efficiently in the presence of porous media with surface catalyzed reaction. Hence, a decline in concentration profile is seen. Figure 12 displays the impact of the melting heat parameter on the axial velocity profile. The escalating axial velocity profile indicates that upon increasing melting heat parameter, molecular motion is enhanced. Figure 13 is sketched for a thermal profile by varying Brownian motion parameter for pseudoplastic fluid. The physical significance of enlargement in the thermal profile is the heating up of the thermal boundary layer by the Brownian motion of nanoparticles. Figure 14 is drawn for altered values of thermophoresis parameter to visualize thermal profile. The upsurge thermal profile for increasing thermophoresis parameter reduces heat exchange in the thermal boundary layer of pseudoplastic fluid. Similarly, Fig. 15 is sketched for concentration profile by taking the variation in thermophoresis parameter. The amount of mass exchange has been reduced causing the escalating thermal profile. Figure 16 depicts the variation in concentration profile by an increase in Schmidt number along with variation in $k_{vs} = 0.5$ to $k_{vs} = 1$. The reaction rate boosts up because of the wider absorption interfacial syrface area on porous media. Additionally, the nanoparticles of reactants become more accelerated and collide much faster than before, causing the thickness of the concentration boundary layer. Therefore, a decline in concentration profile is witnessed. Figure 17 is the depiction of variation in drag force coefficient in the radial direction for non-Newtonian rheology by taking the thickness index of the disk $m = 0.8,1.5,2.5$. Figure 18 represents the drag force coefficient in tangential direction for non-Newtonian rheology by taking the thickness index of disk $m = 0.8,1.5,2.5$. Both figures show the enhancement in drag force coefficient in radial and tangential direction respectively. Heat transfer rate for non- Newtonian rheology is presented in Fig. 19 by escalating thickness index of disk $m = 0.8,1.5,2.5$. The decline in rate of heat transfer is witnessed for the power-law index $n$ throughout 0.3 to 1.2. Figure 20 is the sketch of heat transfer rate for non-Newtonian rheology for mounting values of Prandtl number. Heat transfer rate is seen to increase by increasing Prandtl number. Figure 21 presents the heat transfer rate for increasing value of the power-law index.

Table 1 is developed for numerical values $f^{\prime } (0),\;g^{\prime } (0),\;\theta^{\prime } (0)$ and $- h(\infty )$ for Newtonian fluid with thickness index of disk $m = 0$ and $Ma,K,k_{1} ,k_{2} ,k_{vs} = 0,$ and $\Pr = 2,r = 0.6$. The results obtained are found in excellent agreement with the previous literature. Table 2 signifies the numerical results of $f^{\prime } (0),\;g^{\prime } (0),\;\theta^{\prime } (0)$ and $- h(\infty )$ for varying dimensionless thickness coefficients and power-law index. The results show that variations in the dimensionless thickness coefficient do not make any effect. However, increasing power-law index causing an increase in $f^{\prime } (0),\;g^{\prime } (0),\;\theta^{\prime } (0)$ and $- h(\infty )$.

**Table 1:** Numerical results of $f^{\prime } (0),\;g^{\prime } (0),\;\theta^{\prime } (0)$ and $- h(\infty )$ for Newtonian fluid with thickness index of disk $m = 0$ and $Ma,K,K_{1} ,K_{2} ,k_{vs} = 0$ and $\Pr = 2,r = 0.6$.

Author	$f^{\prime } (0)$	$- g^{\prime } (0)$	$- \theta^{\prime } (0)$	$- h(\infty )$
Andreson^27^	0.51000	0.616000	–	0.883000
Ming^31^	0.51010	0.615591	0.396320	0.882300
Xun^43^	0.51023	0.615921	0.396271	0.884334
Present	0.51023	0.615922	0.396272	0.884333

**Table 2:** Numerical results of $f^{\prime } (0),\;g^{\prime } (0),\;\theta^{\prime } (0)$ and $- h(\infty )$ for increasing power-law index $n$ by taking $\Pr = 2,r = 0.6$ and disk thickness index $m = 0.8$.

$n$	$\alpha$	$f^{\prime } (0)$	$- g^{\prime } (0)$	$- \theta^{\prime } (0)$	$- h(\infty )$
0.8	0.5	0.50244141	0.5527109	0.06664806	0.24911387
	2.0	0.50244141	0.5527109	0.06664806	0.24911387
1.5	0.5	0.58618164	2.5578965	0.57105566	0.13341805
	2.0	0.58618164	2.5578965	0.57105566	0.13341805

## Concluding remarks

The flow of the Ostwald-de-Waele nanofluid over a rotating disk with varying thickness in a spongy medium has been analyzed with melting heat transfer effects. The surface catalyzed reaction is engaged to boosts the chemical reaction as the contact area between reactants and the catalyst increases. This new concept stimulates the reaction rate in comparison to routine homogeneous-heterogeneous reactions. The other novelty of this study is the use of the variable forms of viscosity and thermal conductivity instead of their constant values. The varying values of the power-law index directly affect the fluid viscosity which in turn changes the fluid rheology from pseudo-plastic to dilatant fluid. Similarly, surface catalyzed alters the fluid concentration. The envisaged model is handled with the bvp4c function of the MATLAB software numerically. The graphical illustrations are logically deliberated. The salient findings of this study are:

i. Increasing surface catalyzed parameter causes the decline in concentration profile more efficiently, as it causes a boost in the pace of reaction rate. The reaction rate enhances owing to the broader absorption interfacial area on permeable media. Furthermore, the nanoparticles of reactants become more accelerated and collide much faster than earlier, instigating the thickness of the concentration boundary layer. Therefore, a decrease in concentration profile is observed.
ii. The viscosity function is reliant on the viscous consistency coefficient. Improving power-law index results in the rise in variable viscosity, which in turn liable for the thickening of the boundary layer. Thus, significant augmentation in the flow occurs in the axial direction to compensate for the radial outflow.
iii. The power-law index is more influential than the variable thickness disk index.
iv. Velocity and temperature boundary layer gets thicker for increasing disk thickness index.
v. The concentration profile for pseudoplastic fluid of a chemical species is a decreasing function of Schmidt number.
vi. Larger values of the Prandtl number make the heat transfer process more efficient.
vii. Increasing power-law index leads to monotonic thickening of boundary layers.

## References

[1] Choi SUS (1995). Enhancing thermal conductivity of fluid with nanoparticles. ASME Fluids Eng. Div..

[2] Suleman M, Ramzan M, Ahmad S, Lu D (2019). Numerical simulation for homogeneous–heterogeneous reactions and Newtonian heating in the silver-water nanofluid flow past a nonlinear stretched cylinder. Phys. Scripta.

[3] Tlili I, Ramzan M, Nisa HU, Shutaywi M, Shah Z, Kumam P (2020). Onset of gyrotactic microorganisms in MHD Micropolar nanofluid flow with partial slip and double stratification. J. King Saud Univ. Sci..

[4] Gul N, Ramzan M, Chung JD, Kadry S, Chu YM (2020). Impact of hall and ion slip in a thermally stratified nanofluid flow comprising Cu and Al_2_O_3_ nanoparticles with nonuniform source/sink. Sci. Rep..

[5] Abid N, Ramzan M, Chung JD, Kadry S, Chu YM (2020). Comparative analysis of magnetized partially ionized copper, copper oxide–water and kerosene oil nanofluid flow with Cattaneo-Christov heat flux. Sci. Rep..

[6] Lu D, Ramzan M, ul Huda N, Chung JD, Farooq U (2018). Nonlinear radiation effect on MHD Carreau nanofluid flow over a radially stretching surface with zero mass flux at the surface. Sci. Rep..

[7] Ram P, Kumar V (2014). Heat transfer in FHD boundary layer flow with temperature dependent viscosity over a rotating disk. Fluid Dyn. Mater. Process..

[8] Rashidi MM, Kavyani N, Abelman S (2014). Investigation of entropy generation in MHD and slip flow over a rotating porous disk with variable properties. Int. J. Heat Mass Transf..

[9] Sheikholeslami M, Hatami M, Ganji DD (2015). Numerical investigation of nanofluid spraying on an inclined rotating disk for cooling process. J. Mol. Liq..

[10] Bachok N, Ishak A, Pop I (2011). Flow and heat transfer over a rotating porous disk in a nanofluid. Phys. B.

[11] Kendoush, A. A. Similarity solution for heat convection from a porous rotating disk in a flow field. *J. Heat Trans.*, *135*(8), (2013).

[12] Turkyilmazoglu M (2014). Nanofluid flow and heat transfer due to a rotating disk. Comput. Fluids.

[13] Parsa SM (2021). Reliability of thermal desalination (solar stills) for water/wastewater treatment in light of COVID-19 (novel coronavirus “SARS-CoV-2”) pandemic: What should consider?. Desalination.

[14] Parsa SM, Rahbar A, Koleini MH, Aberoumand S, Afrand M, Amidpour M (2020). A renewable energy-driven thermoelectric-utilized solar still with external condenser loaded by silver/nanofluid for simultaneously water disinfection and desalination. Desalination.

[15] Parsa SM, Yazdani A, Dhahad H, Alawee WH, Hesabi S, Norozpour F, Afrand M (2021). Effect of Ag, Au, TiO_2_ metallic/metal oxide nanoparticles in double-slope solar stills via thermodynamic and environmental analysis. J. Clean. Prod..

[16] Parsa SM, Javadi D, Rahbar A, Majidniya M, Salimi M, Amidpour Y, Amidpour M (2020). Experimental investigation at a summit above 13,000 ft on active solar still water purification powered by photovoltaic: A comparative study. Desalination.

[17] Parsa SM, Javadi D, Rahbar A, Majidniya M, Aberoumand S, Amidpour Y, Amidpour M (2019). Experimental assessment on passive solar distillation system on Mount Tochal at the height of 3964 m: Study at high altitude. Desalination.

[18] Hayat T, Rashid M, Imtiaz M, Alsaedi A (2015). Magnetohydrodynamic (MHD) flow of Cu-water nanofluid due to a rotating disk with partial slip. AIP Adv..

[19] Pordanjani AH, Aghakhani S (2021). Numerical investigation of natural convection and irreversibilities between two inclined concentric cylinders in presence of uniform magnetic field and radiation. Heat Trans. Eng..

[20] Pordanjani AH, Aghakhani S, Afrand M, Mahmoudi B, Mahian O, Wongwises S (2019). An updated review on application of nanofluids in heat exchangers for saving energy. Energy Convers. Manag..

[21] Aghakhani S, Pordanjani AH, Afrand M, Sharifpur M, Meyer JP (2020). Natural convective heat transfer and entropy generation of alumina/water nanofluid in a tilted enclosure with an elliptic constant temperature: Applying magnetic field and radiation effects. Int. J. Mech. Sci..

[22] Yan SR, Aghakhani S, Karimipour A (2020). Influence of a membrane on nanofluid heat transfer and irreversibilities inside a cavity with two constant-temperature semicircular sources on the lower wall: applicable to solar collectors. Phys. Scripta.

[23] Aghakhani S, Ghasemi B, Pordanjani AH, Wongwises S, Afrand M (2019). Effect of replacing nanofluid instead of water on heat transfer in a channel with extended surfaces under a magnetic field. Int. J. Numer. Methods Heat Fluid Flow.

[24] Parsa SM, Rahbar A, Koleini MH, Javadi YD, Afrand M, Rostami S, Amidpour M (2020). First approach on nanofluid-based solar still in high altitude for water desalination and solar water disinfection (SODIS). Desalination.

[25] Mitschka P (1964). Non-Newtonian fluids II. Rotational currents Ostwald-de Waelescher non-Newtonian fluids. Collect. Czechoslovak Chem. Commun..

[26] Mitschka P, Ulbrecht J (1966). Non-Newtonian fluids v frictional resistance of discs and cones rotating in power-law non-Newtonian fluids. Appl. Sci. Res. Sect. A.

[27] Andersson HI, De Korte E, Meland R (2001). Flow of a power-law fluid over a rotating disk revisited. Fluid Dyn. Res..

[28] Attia HA (2003). Unsteady flow of a non-Newtonian fluid above a rotating disk with heat transfer. Int. J. Heat Mass Transf..

[29] Sahoo B (2009). Effects of partial slip, viscous dissipation and Joule heating on Von Kármán flow and heat transfer of an electrically conducting non-Newtonian fluid. Commun. Nonlinear Sci. Numer. Simul..

[30] Ahmadpour A, Sadeghy K (2013). Swirling flow of Bingham fluids above a rotating disk: an exact solution. J. Nonnewton. Fluid Mech..

[31] Griffiths PT (2015). Flow of a generalised Newtonian fluid due to a rotating disk. J. Nonnewton. Fluid Mech..

[32] Griffiths PT, Garrett SJ, Stephen SO (2014). The neutral curve for stationary disturbances in rotating disk flow for power-law fluids. J. Nonnewton. Fluid Mech..

[33] Lin, Y., Zheng, L., & Zhang, X. Magnetohydrodynamics thermocapillary Marangoni convection heat transfer of power-law fluids driven by temperature gradient. *J. Heat Trans.*, *135*(5), (2013).

[34] Sui J, Zheng L, Zhang X, Chen G (2015). Mixed convection heat transfer in power law fluids over a moving conveyor along an inclined plate. Int. J. Heat Mass Transf..

[35] Zheng L, Lin Y, Zhang X (2012). Marangoni convection of power law fluids driven by power-law temperature gradient. J. Franklin Inst..

[36] Ming C, Zheng L, Zhang X (2011). Steady flow and heat transfer of the power-law fluid over a rotating disk. Int. Commun. Heat Mass Transfer.

[37] Shufrin I, Eisenberger M (2005). Stability of variable thickness shear deformable plates first order and high order analyses. Thin Walled Struct..

[38] Fang T, Zhang J, Zhong Y (2012). Boundary layer flow over a stretching sheet with variable thickness. Appl. Math. Comput..

[39] Subhashini SV, Sumathi R, Pop I (2013). Dual solutions in a thermal diffusive flow over a stretching sheet with variable thickness. Int. Commun. Heat Mass Trans..

[40] Hayat T, Farooq M, Alsaedi A, Al-Solamy F (2015). Impact of Cattaneo-Christov heat flux in the flow over a stretching sheet with variable thickness. Aip Adv..

[41] Abdel-Wahed MS, Elbashbeshy EMA, Emam TG (2015). Flow and heat transfer over a moving surface with non-linear velocity and variable thickness in a nanofluids in the presence of Brownian motion. Appl. Math. Comput..

[42] Li B, Chen X, Zheng L, Zhu L, Zhou J, Wang T (2014). Precipitation phenomenon of nanoparticles in power-law fluids over a rotating disk. Microfluid. Nanofluid..

[43] Xun S, Zhao J, Zheng L, Chen X, Zhang X (2016). Flow and heat transfer of Ostwald-de Waele fluid over a variable thickness rotating disk with index decreasing. Int. J. Heat Mass Transf..

[44] Hayat T, Rashid M, Khan MI, Alsaedi A (2018). Melting heat transfer and induced magnetic field effects on flow of water based nanofluid over a rotating disk with variable thickness. Res. Phys..

[45] Chaudhary MA, Merkin JH (1995). A simple isothermal model for homogeneous-heterogeneous reactions in boundary-layer flow. I. Equal diffusivities. Fluid Dyn. Res..

[46] Ramzan M, Riasat S, Chung JD, Chu YM, Sheikholeslami M, Kadry S, Howari F (2021). Upshot of heterogeneous catalysis in a nanofluid flow over a rotating disk with slip effects and Entropy optimization analysis. Sci. Rep..

[47] Doh DH, Muthtamilselvan M, Swathene B, Ramya E (2020). Homogeneous and heterogeneous reactions in a nanofluid flow due to a rotating disk of variable thickness using HAM. Math. Comput. Simul..

[48] Liu C, Pan M, Zheng L, Lin P (2020). Effects of heterogeneous catalysis in porous media on nanofluid-based reactions. Int. Commun. Heat Mass Trans..

[49] Hayat T, Haider F, Muhammad T, Ahmad B (2019). Darcy-Forchheimer flow of carbon nanotubes due to a convectively heated rotating disk with homogeneous–heterogeneous reactions. J. Therm. Anal. Calorim..

